# Scaffold Hopping with Generative Reinforcement Learning

**DOI:** 10.1021/acs.jcim.5c00029

**Published:** 2025-06-26

**Authors:** Luke Rossen, Finton Sirockin, Nadine Schneider, Francesca Grisoni

**Affiliations:** † Institute for Complex Molecular Systems (ICMS), Department of Biomedical Engineering, 3169 Eindhoven University of Technology, P.O. Box 513, 5600 MB Eindhoven, The Netherlands; ‡ 98560 Novartis Biomedical Research, Novartis Campus, 4002 Basel, Switzerland; § Centre for Living Technologies, Alliance TU/e, WUR, UU, UMC Utrecht, Princetonlaan 6, 3584 CB Utrecht, The Netherlands

## Abstract

Scaffold hopping–the design of novel scaffolds for existing lead candidates–is a multifaceted and nontrivial task, for medicinal chemists and computational approaches alike. Generative reinforcement learning can iteratively optimize desirable properties of *de novo* designs, thereby offering opportunities to accelerate scaffold hopping. Current approaches confine the generation to a predefined molecular substructure (e.g., a linker or scaffold) for scaffold hopping. This confined generation may limit the exploration of the chemical space and require intricate molecule (dis)­assembly rules. In this work, we aim to advance reinforcement learning for scaffold hopping, by allowing “unconstrained”, full-molecule generation. This is achieved via the **RuSH** (**R**einforcement Learning for **U**nconstrained **S**caffold **H**opping) approach. RuSH steers the generation toward the design of full molecules having a high three-dimensional and pharmacophore similarity to a reference molecule, but low scaffold similarity. In this first study, we show the flexibility and effectiveness of RuSH in exploring analogs of known scaffold-hops and in designing scaffold-hopping candidates that match known binding mechanisms. Finally, the comparison between RuSH and two established methods highlights the benefit of its unconstrained molecule generation to systematically achieve scaffold diversity while preserving optimal three-dimensional properties.

## Graphical abstract

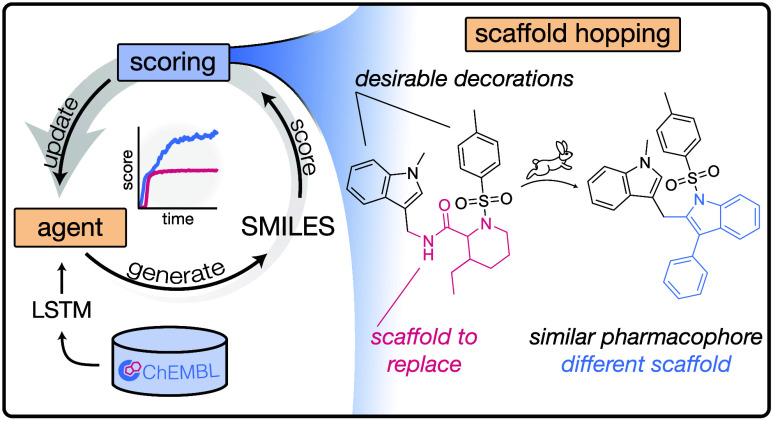

## Introduction

In recent years, generative deep learning has shown its promise for *de novo* drug design.^1–6^ Generative approaches can explore the chemical space and produce molecules with desirable properties on-demand.^7^ Among the existing generative deep learning paradigms, reinforcement learning is particularly attractive to “navigate” multiple structure–activity/property relationships in unison, which is oftentimes nontrivial.^8^ This is achieved by iteratively updating a model based on the rewards of an external scoring function, designed to optimize one or more desirable molecular properties.^9,10^ One particularly interesting, and relatively unexplored, application of reinforcement learning is “scaffold hopping”.^8^ Scaffold hopping refers to finding isofunctional molecular structures with different cores,^8,11^ aiming to improve certain compound properties (e.g., selectivity, synthetic accessibility, absorption, distribution, metabolism, excretion, and toxicity), or lead to new patentable structures.

Several studies have focused on scaffold hopping with generative deep learning.^1,12–18^ Most available approaches cast scaffold hopping as a conditional linker/scaffold generation problem, aiming to connect a set of predefined decorations via dedicated substructures.^12–15^ While promising for fragment prioritization,^15,19^ such approaches constrain the molecular generation to predefined portions of a molecule only. This is typically achieved by rule-based data prepreparation,^12–15^ or additional modeling steps.^1,16–18^ These aspects might lead to a limited exploration of the chemical space, a suboptimal matching of desirable three-dimensional and pharmacophore properties, or chemically “unintuitive” structures depending on the rules used. In this context, an “unconstrained” molecular generator could alleviate these concerns, and bears promise to advance scaffold hopping. Recent advancements in scoring function design are promising,^10^ but lack dedicated functionality for scaffold hopping.

Stemming from these considerations, in this work we leverage reinforcement learning for scaffold hopping via “unconstrained” molecule generation for the first time. Our approach–Reinforcement Learning for Unrestricted Scaffold Hopping (RuSH, Figure 1)–aims to generate molecules that have (a) high three-dimensional and pharmacophore similarity to a given reference molecule, to preserve the ligand binding affinity to a target,^16^ and (b) low scaffold similarity in terms of substructures present in the molecule. This was achieved by combining full-molecule generation with a new scoring function, specifically designed for scaffold hopping. Several studies have, in fact, shown the key role of scoring functions to steer generative reinforcement learning toward promising solutions.^9,15,24,25^ To this extent, RuSH relies on the well-established reinforcement learning framework REINVENT.^9^

**Figure 1:**
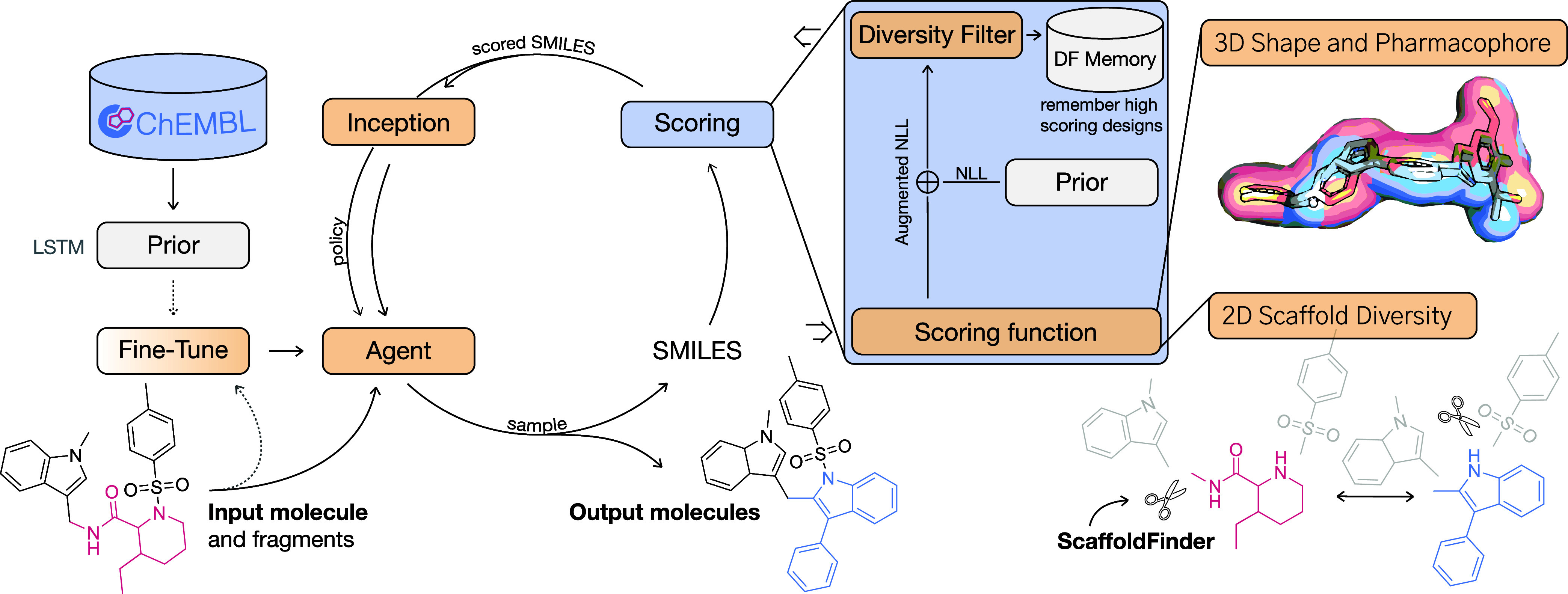
RuSH (Reinforcement Learning for Unconstrained Scaffold Hopping). A generator (long short-term memory network, LSTM) is trained on ChEMBL^20,21^ (Prior), and subsequently fine-tuned by transfer learning on a single reference molecule. The Prior will act as the reinforcement learning agent, and at each epoch, SMILES strings are sampled from the model. These strings are scored by the scoring function, which rewards for (a) high three-dimensional and pharmacophore similarity (via ROCS^22^) and low scaffold similarity^23^ to the same reference molecule. To detect scaffolds in generated designs, we developed an algorithm, ScaffoldFinder. During a reinforcement learning run, a diversity filter (DF) ‘memory’ is used to remember all high-scoring designs. An augmented Negative Log-Likelihood (NLL) is computed to update the policy of the agent across cycles. Inception^9^ is used to speed up the reinforcement learning process by periodically exposing the agent to high scoring designs of earlier epochs.

In this first study, RuSH was applied to four protein targets with reported scaffold-hops. The approach was evaluated for its learning behavior, its capacity to retrieve known scaffold-hops, and to explore new scaffold hopping candidates. Moreover, it was compared with two state-of-the-art scaffold hopping approaches (DeLinker^12^ and Link-INVENT^15^). The results show the potential of RuSH for scaffold hopping, and underpin the advantages of full-molecule generation in terms of molecular quality, score optimization and learning strategies such as fine-tuning.

## Results and Discussion

### Scaffold Hopping with Reinforcement Learning

Given a reference bioactive molecule, RuSH aims to design molecules that (a) conserve three-dimensional shape and pharmacophoric properties (relevant for binding to macromolecular targets^26^), and (b) possess a different scaffold than the reference molecule. To this end, we devised a scoring function that jointly rewards for (a) three-dimensional similarity, and (b) scaffold substructure dissimilarity. In what follows, after introducing the rationale of the novel scoring function, we outline its integration with reinforcement learning.

#### Scoring Function

We devised an *ad-hoc* scoring function to capture both two-dimensional (2D) dissimilarity (in terms of shared scaffold substructures) and three-dimensional (3D) similarity (in terms of shape and pharmacophore) between a *de novo* design and a reference molecule. This is achieved by combining two separate rewards, in three stages:

- *2D reward.* To capture the scaffold (dis)­similarity in terms of substructures, the designed molecules are analyzed for their inclusion of decorations, and their scaffolds compared to that of the reference molecule. To this end, we devised the ScaffoldFinder algorithm (Figure 2), which searches for the correct inclusion of a list of reference decorations in a *de novo* design (via maximum common substructure matching^27^), and allows a degree of “fuzziness” in the comparison to allow for generative exploration (as parametrized via α, eqs 1 and 2). Only if a *de novo* design contains all decorations, its scaffold is compared with the one of the reference molecule. The 2D reward is then computed via the Tanimoto distance (eq 3) on Extended Connectivity Fingerprints (ECFPs)^28,29^ between the respective scaffolds (the higher the distance, the higher the reward).
- *Partial reward.* To address sparse reward problems typical of reinforcement learning,^2,30^ a partial reward (of 0.3) is given to designs that contain some, but not all decorations, determined by the ScaffoldFinder algorithm. These molecules are then also omitted from further 3D scoring, optimizing the scoring function for compute time. This aspect both expedites the reinforcement learning campaign, and improves the ability of models to learn the inclusion of all decorations correctly.
- *3D reward.* For each design, an ensemble is generated of geometry-optimized conformers (up to 32 conformers per enumerated stereoisomer) using OMEGA.^31^ Each conformer is compared with the crystallographic pose of the reference molecule within the macro-molecular target (protein) of interest, as obtained from the Protein Data Bank.^32^ The alignment and comparison is achieved using Rapid Overlay of Chemical Structures (ROCS^22^), which uses volumetric shape and pharmacophore similarity matching for alignment and scoring (“shape” and “color” scores, respectively). Scores are computed for each conformer (as a weighted average between ROCS shape and color scores, eq 5). For the 3D reward, the design is assigned the score of its highest-scoring conformer, we consider this a “greedy” approach (no hits lost).

**Figure 2:**
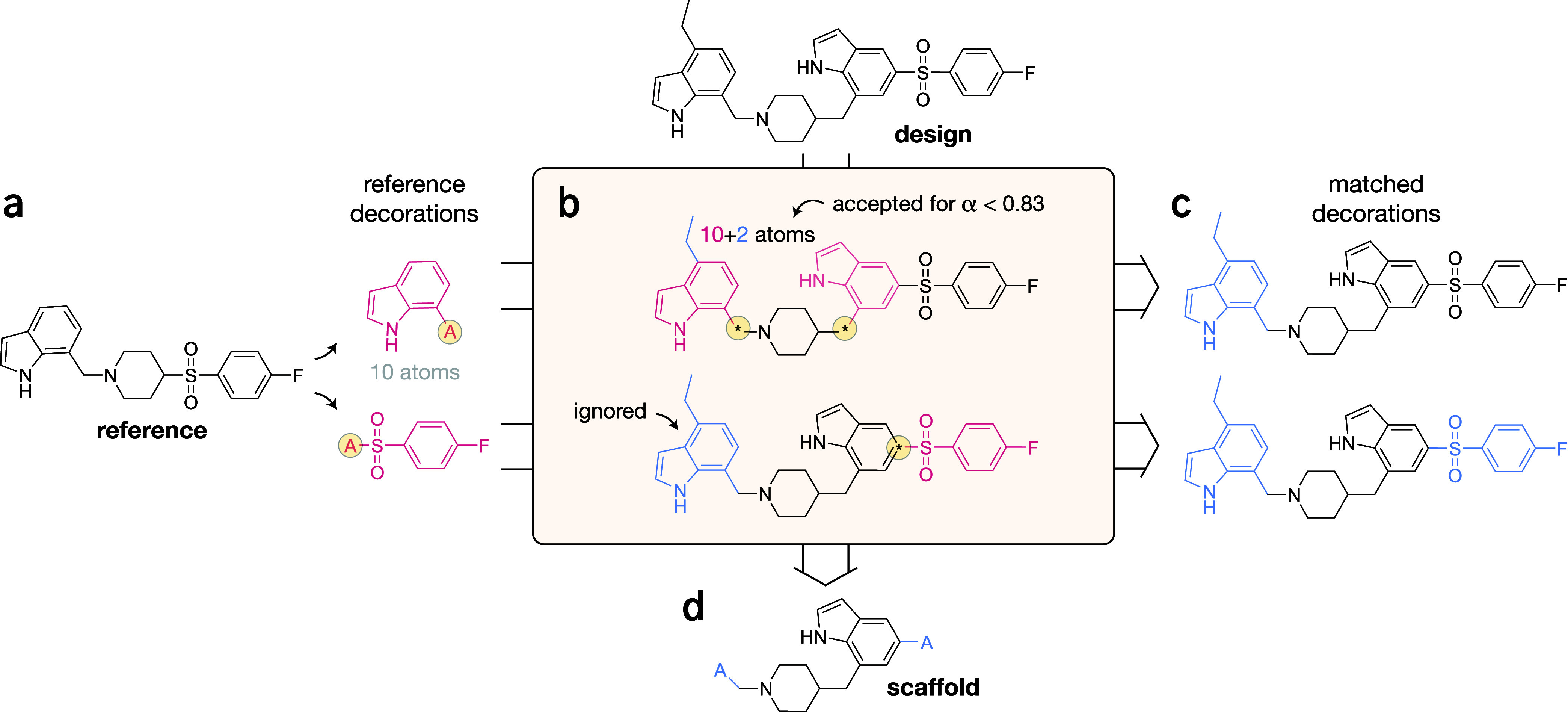
ScaffoldFinder algorithm. Given a reference molecule and a set of decorations (a), ScaffoldFinder performs “fuzzy matching” (using a parameter called α) with a *de novo* design (b), to identify a set of corresponding decorations (c). If all decorations have been matched, they are cleaved and the scaffold is returned (d). α may range from 0.0 to 1.0, and determines how much the decoration may vary from the reference decoration in terms of atom count.

Finally, the 2D and 3D rewards are combined via a weighted harmonic mean for a final score (eq 6), such that optimizing for one reward cannot go at the cost of another. This reward is then used to steer the reinforcement learning agents toward promising regions in the chemical space.

#### Reinforcement Learning Framework

RuSH relies on REINVENT,^9^ a well-established reinforcement learning framework that allows for unconstrained molecule generation. Our pipeline consist of the following steps (Figure 1):

(1) *Molecule generator*. Long–short-term memory (LSTM) networks^33^ were used to learn from (and generate) molecules as Simplified Input Line Molecular Entry Systems (SMILES) strings.^34^ We considered two generative models (termed “Priors”) per experiment: (a) an LSTM trained on 1.5 M drug-like molecules from ChEMBL,^20,35^ and (b) a “fine-tuned” LSTM, obtained by subsequently transfer learning the ChEMBL Prior with the SMILES of the reference bioactive molecule.
(2) *Agent*. Reinforcement learning “agents” perform specific actions to learn how to achieve a specific goal. Here, the chosen Prior will act as the initial agent, and reinforcement learning begins. The action in this context is defined as generating a series of SMILES strings (64 in this work) at each reinforcement learning cycle, or “epoch”.
(3) *Scoring*. All SMILES generated by the agent are scored using the novel scoring function. A diversity filter^2,9^ checks and penalizes designs whose Bemis-Murcko scaffold^36^ has been generated too many times (more than 30 in this work). The diversity filter “memory” also stores all high-scoring designs (above 0.4 in this work) in a reinforcement learning run. These designs are considered the generated designs of a given reinforcement learning run.
(4) *Policy Evaluation and Improvement*. The awarded scores are used to update the agent for the next iteration. At every epoch, the agent is evaluated for (a) the quality of the designs (via the scoring function), and (b) their “chemical plausibility”. This is estimated by obtaining the negative log-likelihood (NLL) of the generated SMILES, as predicted by the original Prior model before reinforcement learning.^9^ These two aspects are then combined by computing what is called the “Augmented NLL” ^9,37^ (eq 7), which is used as the loss to update the policy of the agent (eq 8), and steer the generation toward optimal solutions.
(5) *Inception.* REINVENT’s inception feature is used to periodically expose the agent to previously generated, high scoring, SMILES strings, to accelerate the navigation toward desired regions in chemical space.^9^

### Scaffold Hopping Case Studies

As a proof-of-concept, we applied RuSH to four protein targets (Figure 3): (a) Proviral Insertion Site of Moloney Murine Leukemia 1 Kinase (PIM1), (b) Human Immunodeficiency Virus 1 protease (HIV1), (c) c-Jun N-terminal kinase 3 (α1) (JNK3), and (d) Soluble Adenyl Cyclase (ADCY10). For each target, we selected one scaffold hopping case reported in literature (Figure 3), based on the availability of two ligands with different scaffolds and near-identical decorations, and whose crystal structures were available on the PDB.^32^ One molecule was used as the reference, and the other one as a ground truth target for scaffold hopping. RuSH was evaluated for its ability to retrieve the known scaffold-hops, as well as produce designs that constitute good scaffold hopping options. In what follows, we discuss the reinforcement learning behavior of RuSH, as well as the recovery of known scaffold-hops and its performance in generating suitable alternatives.

**Figure 3:**
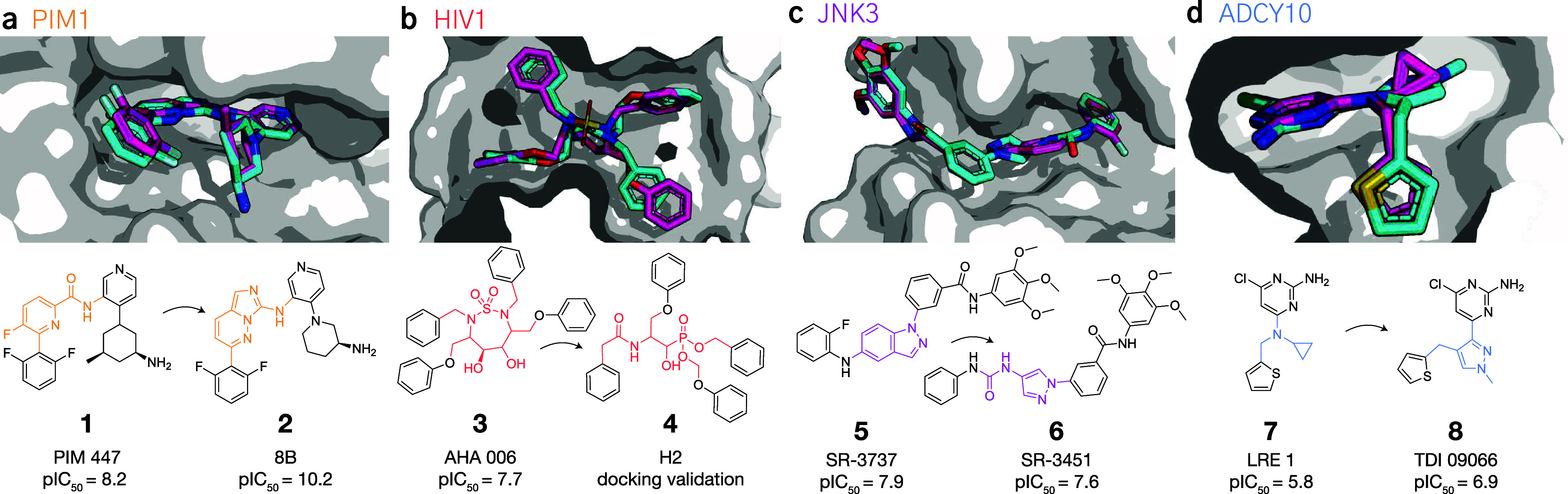
Selected scaffold hopping cases. (a) PIM1. Reference molecule **1** (PDB-ID: 5DWR) and scaffold-hop **2** (PDB-ID: 5KZI).^38^ (b) HIV1, **3** (PDB-ID: 1AJV) and scaffold-hop **4** (computational validation^39^). Superimposed instead is AHA-001 (PDB-ID: 1AJX) in the HIV1 active site. (c) JNK3, **5** (PDB-ID: 3FI3) and hop **6** (PDB-ID: 3FI2),^40^ (d) ADCY10, **7** (PDB-ID: 5IV4) and scaffold-hop **8** (PDB-ID: 8CO7).^41^ The pose of the reference molecule and the scaffold-hop in the binding pocket are depicted in magenta and cyan, respectively. The 2D depictions highlight the scaffold diversity with colors.

#### Learning Behavior

For each target, we analyzed the effect of transfer learning on the agent’s capacity to include the input decorations, explore novel scaffolds, and reaching optimal values of the scoring function (Figure 4). Moreover, we monitored the speed of the agent across reinforcement learning epochs, as a function of the progressive inclusion of all reference decorations (Supporting Figure S1).

**Figure 4:**
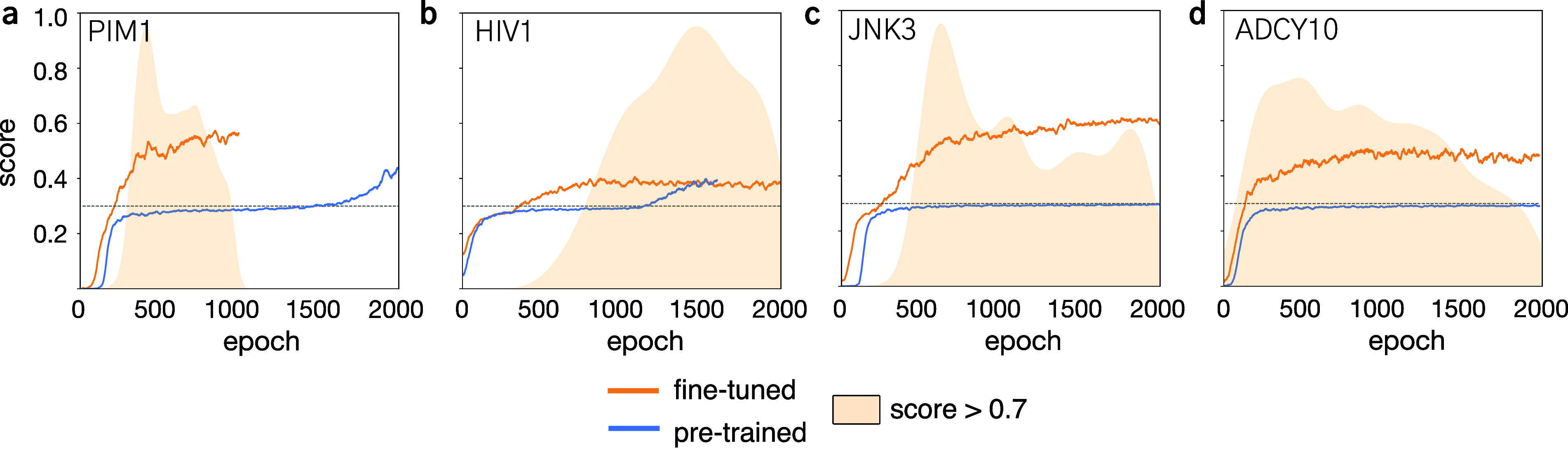
Reinforcement learning experiments for the chosen protein targets. (a) PIM1, (b) HIV1, (c) JNK3, and (d) ADCY10. For each target we compare the results of the ChEMBL Prior (Pretrained) with the fine-tuned Prior using the reference molecule (solid line). The average score per epoch (64 SMILES sampled per epoch) is depicted. The normalized distribution plots for generated designs that scored higher than 0.7 is also reported to highlight when high scoring designs are generated.

Across all experiments, the agents initialized with transfer learning demonstrated a faster inclusion of the decorations (Figure 4 and Supporting Figure S1). This is particularly evident with structurally complex decorations (i.e., JNK3 and ADCY10, Figure 4c,d), where transfer learning was fundamental to achieve the decoration inclusion (corresponding to a score above 0.3). The partial reward has a beneficial effect to save compute time when the designs do not correctly contain the decorations (Supporting Figure S1), allowing to quickly reach the point where decoration inclusion is learned and the average score increases (Figure 4 and Supporting Figure S1). Transfer learning consistently led to higher cumulative rewards (Figure 4), owed to an earlier inclusion of decorations, while it did not affect the convergence to a stable score. No consistent patterns were observed across case studies when monitoring the generation of high-scoring designs (above 0.7). Each case study showed unique trends. The diversity filter played a major role in chemical navigation, forcing the agent to explore new designs when too many similar (high-scoring) designs were generated. For example, in the ADCY10 case study, we observed a decrease in the overall number of designs scoring above 0.7, but an increase in designs scoring above 0.9 (Supporting Figure S1d).

Finally, transfer learning did not affect the generation of unique designs (Figure 5). Moreover, it yielded no remarkable difference in the frequently generated Bemis–Murcko scaffolds^36^ per target (Figure 5). For instance, on PIM1 and HIV1, the two agents shared more than half of their most common scaffolds (50 and 60%, respectively, Figure 5). These findings align with existing literature,^9^ and suggest that transfer learning is beneficial for accelerating the navigation of chemical space.

**Figure 5:**
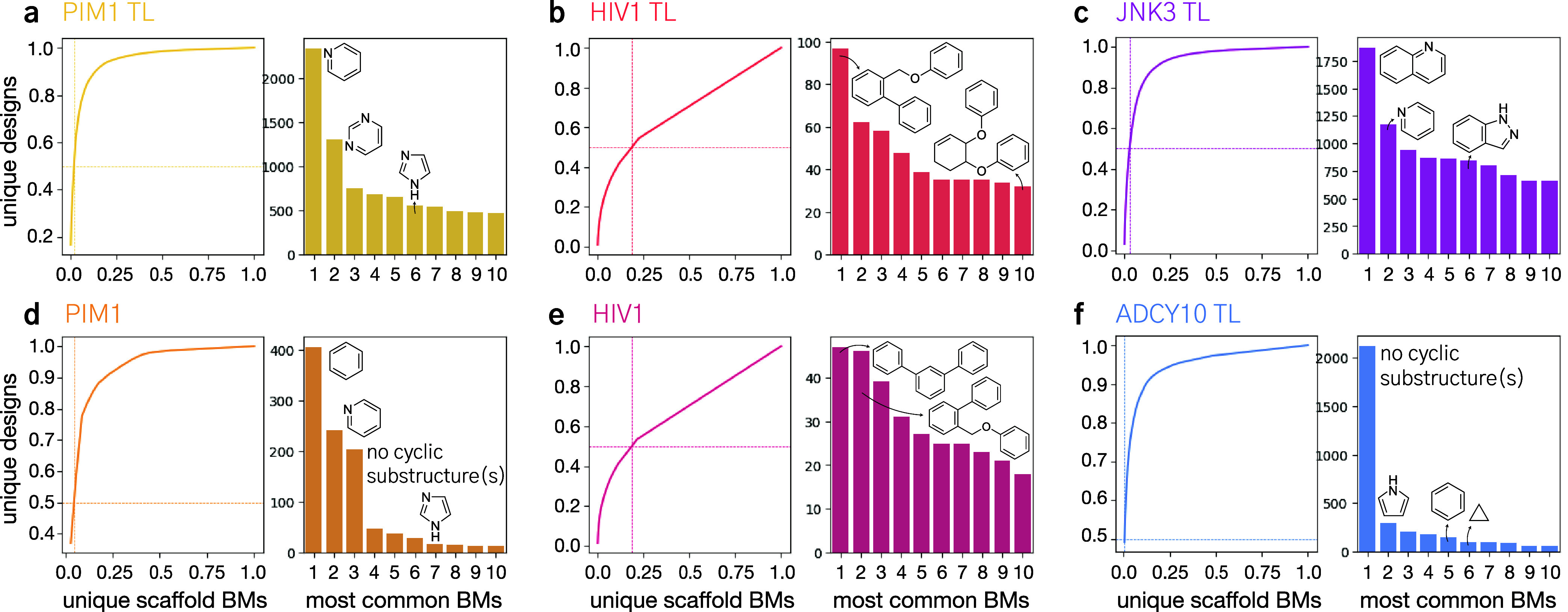
Scaffold diversity analysis. Scaffold diversity analysis across targets and training strategies. For each target, we depict the “retrieval curves”, which depict the fraction of unique Bemis–Murcko (BM) scaffolds versus the fraction of generated designs that contain them (left). Moreover, we report the frequency of the 10 most common BM scaffolds (right). For each target, we analyzed agent trained only on ChEMBL (a–c, f), as well as agents fine-tuned on the considered macromolecular target (d, e). The following targets are considered: PIM1 (a, d), HIV1 (b, e), JNK3 (c), ADCY10 (f). ChEMBL agents for JNK3 and ADCY10 did not produce designs that scored above 0.4 and are hence omitted.

#### Recovering Known Scaffold-Hops

No agent recovered the known scaffold-hop exactly. We analyzed the generated designs for their structural similarity to the known scaffold-hops (Figure 3). A *t*-distributed stochastic neighbor embedding (*t*-SNE^42^) was used to inspect the generative landscape, and select compounds similar to the known scaffold-hops (Supporting Figure S7).

In three out of four targets, RuSH produced highly similar designs to the known scaffold-hops (Figure 6), i.e., for HIV1 (known: **4**, designed: **9**), JNK3 (known: **6**, designed: **10**), and ADCY10 known: **8**, designed: **11**. No molecule similar to the known scaffold-hop for PIM1 (**2**) was recovered, as the agents instead favored designs with singular, nonfused rings.

**Figure 6:**
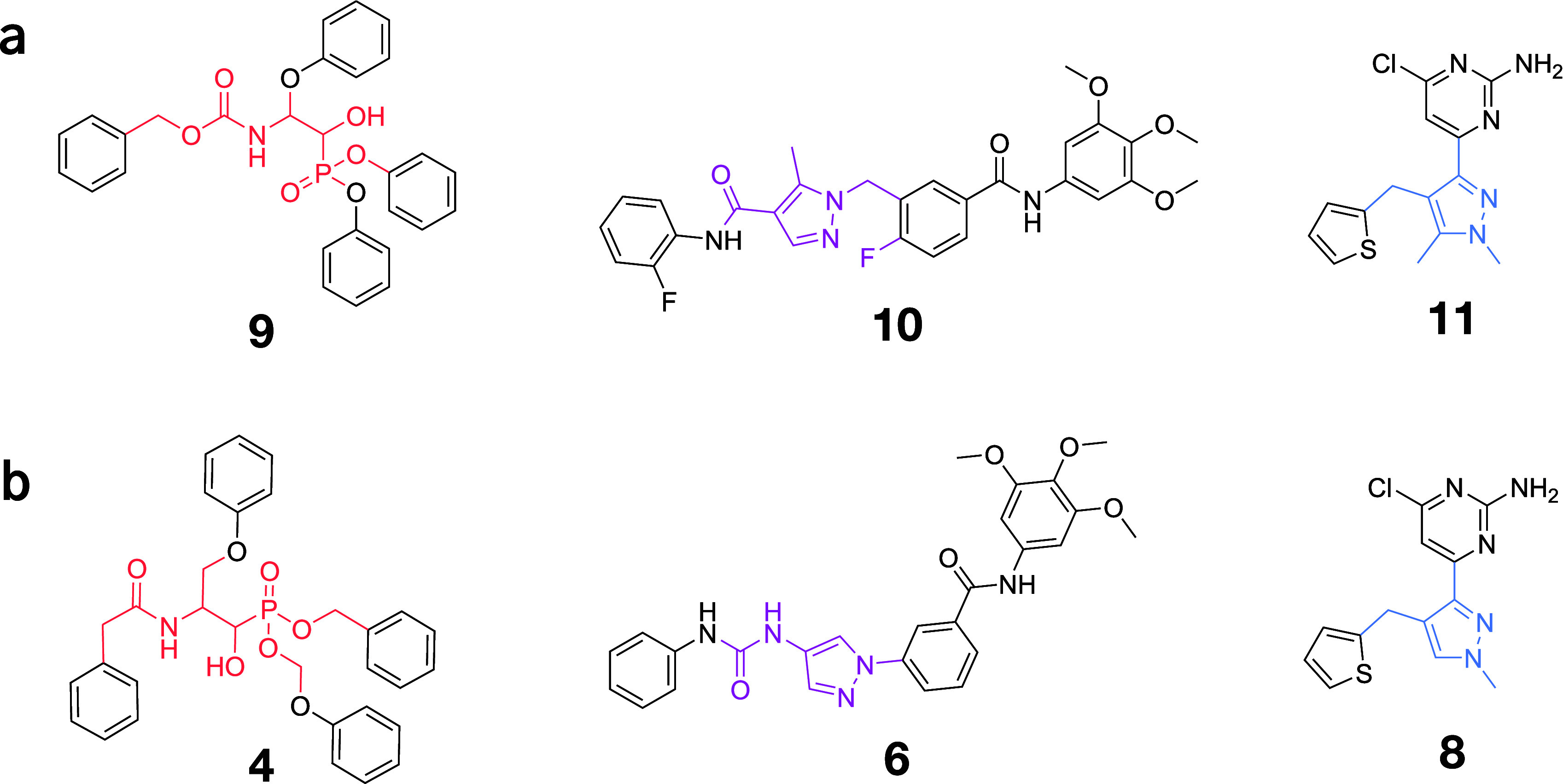
Highest-scoring most-similar designs to known hops. For each case study, we report the high-scoring designs (a) that were structurally similar to the known scaffold-hops (b). HIV1: scaffold-hop **4** in comparison with design **9** (score = 0.60). JNK3: scaffold-hop **6** in comparison with design **10** (score = 0.67). ADCY10: scaffold-hop **8** in comparison with design **11** (score = 0.78). No design similar to the known scaffold-hop for PIM1 (**1**) was designed.

Overall, our scoring function served as a good proxy to steer the design toward the known scaffold-hops. In addition to the selected designs, several high-scoring molecules had a high structural similarity to the scaffold-hops (Supporting Figure S7). These observations suggest the ability of our scoring function to steer the model toward relevant regions of the chemical space. We acknowledge the limitation of this analysis, notably that many scaffold-hopping ground truths may exist for a given reference molecule, including many, as of now undiscovered designs.

#### Discovering New Scaffold-Hops

All the generated designs were selected for an in-depth analysis on their suitability as novel scaffold hopping candidates. After strictly filtering for several desirable properties (via Novartis in-house models, see Materials and Methods section), filtered designs were docked in the binding pocket of the corresponding macro-molecular target using Glide^43^ and checked for quality with HYDE.^44^ Their poses were filtered and ranked based on the docking quality (see Materials and Methods section), and the remaining top candidates were visually inspected for their interactions with the binding pocket.

Between 0.4 and 2.5% of the *de novo* designs were retained per target (Supporting Table S10). Transfer learning yielded a higher number of retained molecules across all targets (Supporting Table S10), especially visible for HIV1, where all the designs obtained using the ChEMBL Prior were filtered out. For each target, we selected five molecules corresponding to known binding modes from literature (Figure 7, designs **12**–**29**), and compared them with the crystallographic pose of the reference molecules.

- PIM1 (Figure 7a). Designs **12**–**16** match known relevant interactions, i.e., in terms of hydrophobic pocket coverage^38^ and polar interactions (with Asp128 and Glu171 ^45^). Most designs contained four- and five-membered aromatic heterocycles, consistently with literature.^38,45^ Interestingly, thiazole scaffolds (**15**) were also generated, resulting in designs similar to known PIM1 inhibitors.^45^ Thanks to the unconstrained generation, variations of the input decorations were generated (in a controlled fashion), leading to structurally novel designs such as **16**–where the cyclohexyl decoration of **1** is exchanged for a fused cyclopentane-tetrahydrofuran, while preserving a high pose similarity.
- HIV1 (Figure 7b). Designs **17**–**20** are within range to interact via scaffold with Ile50(’) and Asp25(’),^46^ except for **17** which lacks a hydrogen accepting group for interactions with Asp25(’). For all designs, the decorations are positioned in high agreement with **3**. These designs show various cyclo-alkyl scaffolds, with alternative functional groups for hydrogen bonding compared to **3**. Design **18** resembles the scaffold of AHA-001,^46^ an urea analogue of **3** (Figure 3b).
- JNK3 (Figure 7c). Designs **22**-**24** were predicted to form relevant interactions with Met149.^40^ The structural space surrounding **6** was well populated (Supporting Figure S7), although many were filtered by 3D postprocessing for torsion quality (as the known scaffold-hop itself). We note design **24**, one of high similarity to the ground truth **6**.
- ADCY10 (Figure 7d). Designs **25**-**29** interact with relevant residues (Val167 and Met337 ^41,47^ ) and show general agreement with the pose of the known scaffold-hop **8**. **25**, **26** and **29** feature aromatic heterocycle scaffold—similar to **8**—with better exit vectors than the reference molecule (**7**).^41^ Designs **27** and **28** offer further alternatives for scaffold hopping with chemistry different from both **7** and **8**.

**Figure 7:**
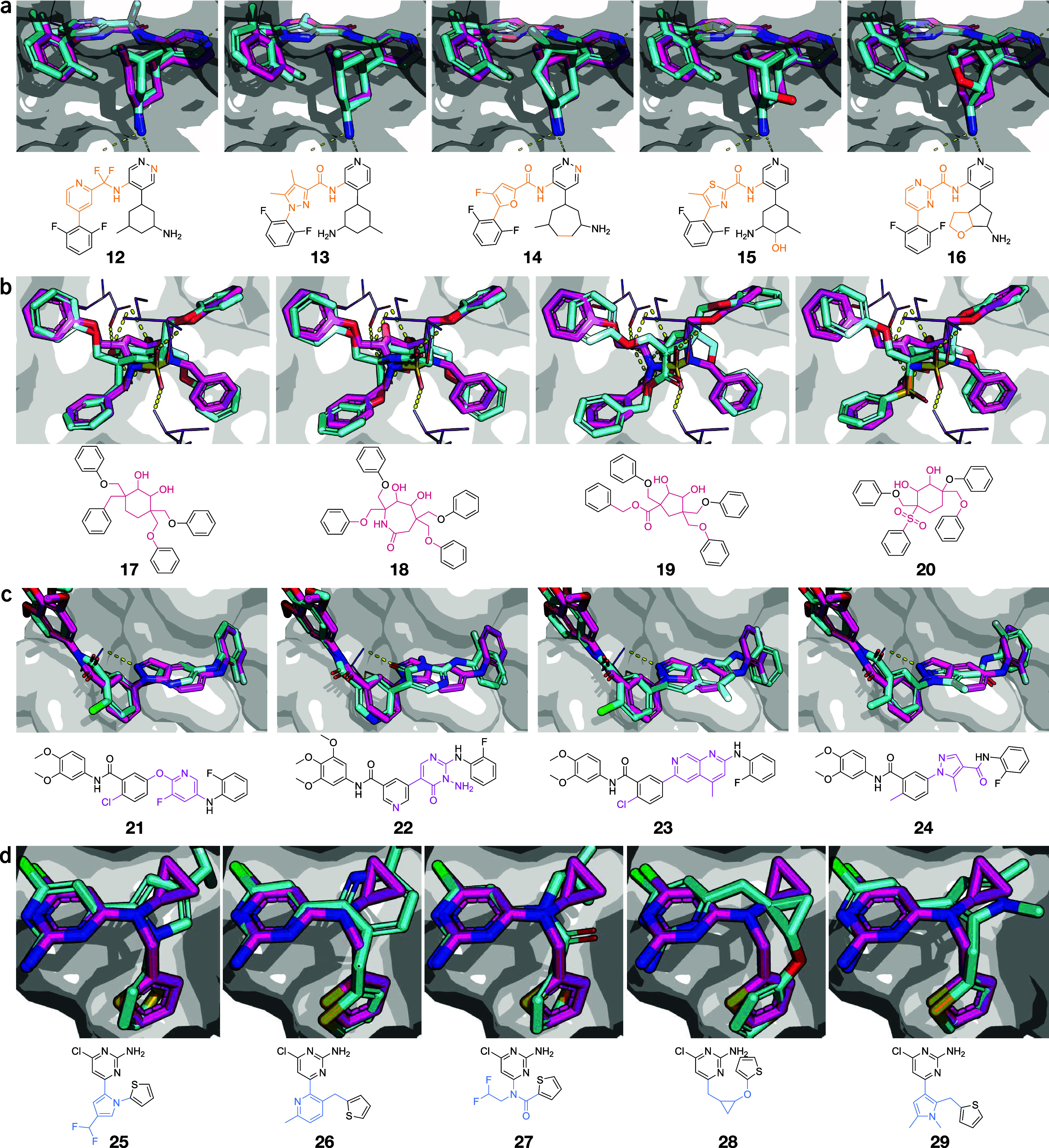
Designed scaffold-hops compared with the bioactive reference. (a) PIM1; (b) HIV1; (c) JNK3; and (d) ADCY10. The docked poses for each design (as obtained from Glide,^43^ cyan) are compared with the crystallographic pose of the respective reference molecule, depicted in magenta (PIM1: **1** and PDB-ID = 5DWR; HIV1: **3** and PDB-ID = 1AJV; JNK3: **5** and PDB-ID = 3FI3; ADCY10: **7** and PDB-ID = 5IV4). In the 2D depictions, the scaffold variations of the designs compared to the reference molecule are highlighted with colors.

These results show that RuSH bears promise to produce designs that contain new molecular scaffolds, while retaining high three-dimensional and pharmacophore similarity. Moreover, thanks to the unconstrained generation, scaffold hopping designs can contain an arbitrary number of decorations, and can offer structural variations of the decorations (i.e., as parametrized by the α value in ScaffoldFinder). A detailed posthoc study (see Supporting Figure S10) reveals a range of values for α reliably produce scaffold hopping designs across case studies, without detriment to model performance or scoring. For a complete overview of designs **12**–**29** we refer to Supporting Figure S8.

### Benchmarking

We compared the RuSH with two well-established methods: (a) Link-INVENT,^15^ which expands REINVENT for fragment-conditioned design of scaffolds (originally referred to as “linkers”), and (b) DeLinker,^12^ which leverages 3D information through a graph-based generative model for scaffold (linker) design. For RuSH, we only considered fine-tuned agents owed for their ability to reliably produce scaffold-hopping designs. To better inspect the effect of our scoring function, it was also applied to Link-INVENT. Each RL approach was run for 1000 cycles, and all designs that scored above 0.4 were stored. DeLinker was sampled in the same order of magnitude as the number of designs produced by RuSH. Generated designs were evaluated for the following properties (Table 1):

- *Generative ability*. We report the number of valid generated designs (such as SMILES corresponding to “chemically valid” molecules), and uniqueness (frequency of non structurally redundant molecules among the designs). Although these metrics are susceptible to trivial baselines,^48^ they offer insights into a generative models capacity to produce a correct molecular SMILES or graphs (DeLinker). For RL methods, these numbers are also indicative of the ability to produce well-scoring designs. All methods produced a sizable number of valid and unique designs. The only exception being DeLinker and Link-INVENT for HIV1. Both methods failed to successfully generate designs connecting all four decorations. This case study required the connection of four decorations, whereas these literature methods are limited to just two. Based on the training data preparation methods, we also suspect the reference molecule is far out of distribution for both methods. For RuSH (REINVENT), a significant portion of the reinforcement learning runs were spent learning the inclusion of the decorations, resulting in 10–16% of designs scoring above 0.4. For RuSH (LinkInvent), this was 80–90% omitting the HIV1 case.
- *Molecular quality.* We measured the suitability of the designs to be considered as good candidates for drug discovery. Designs were filtered (a) using DeLinker’s postprocessing,^12^ which captures basic decoration inclusion, the presence of undesirable substructures, and correct ring aromaticity (see Materials and Methods section), and (b) for their correct inclusion of all decorations using ScaffoldFinder. In general, the number and percentage of retained molecules varied on a target- and method-basis (Table 1). RuSH consistently produced a higher percentage of “high quality” designs (the highest for HIV1, ADCY10 and JNK3). For HIV1, RuSH was the only method able to reliably produce numerous high quality designs (2294 (37%)), followed by Link-INVENT (91 (00%)). These results highlight a potential advantage of using full-molecule design over linker-based methods, which are typically limited to a fixed number of decorations. For both LinkInvent and DeLinker, this was just two decorations. The ability to easily perform transfer learning in the unconstrained setting further highlights an advantage, when the reference structure is potentially out of distribution for the Prior.
- *Molecular distance and scaffold diversity*. We evaluated the methods in terms of their capacity to produce structurally diverse candidates for scaffold hopping, in terms of their full structure and their scaffold specifically. To this end, we measured (a) the global and scaffold molecular distance, as the Jaccard^23^ distance (eq 3) to the reference molecule and its scaffold, and (b) the Scaled Shannon Entropy (SSE) of the scaffolds,^49^ which captures the scaffold heterogeneity within a molecular set (SSE = 0 indicates that all scaffolds share the same cyclic substructure(s), and SSE = 1 indicates that all scaffolds are unique). For SSE, the Bemis–Murcko algorithm was used to retrieve the cyclic substructures of the scaffolds.^36,50^ No method was consistently better on all metrics. Our scoring function showed the highest SSE values in three out of four cases when used within RuSH (HIV1, JNK3) or Link-INVENT (PIM1 and JNK1). On these targets, it also showed the highest molecular distances. We observed the same trends in diversity for the subset of designs that scored high on both 2D and 3D metrics. Linker-based methods were more likely to generate cyclic substructures compared to unconstrained generation, which was particularly apparent for ADCY10 designs (Table 1). Here, RuSH produced ca. 50% scaffolds without cyclic substructures. By our analysis, these are all grouped under the same “no cyclic substructure(s)”, lowering the SSE metric. All methods succeeded at generating designs whose scaffolds had a low similarity to the reference molecule. RuSH showed the lowest scaffold distance for PIM1 (average Jaccard distance of 0.77 ± 0.06) and the highest for ADCY10 (0.92 ± 0.05). Lastly, we canonicalized all generated designs and compared their overlap between methods. In most pairwise comparisons, less than 1% of designs were shared. Notable exceptions occurred only for ADCY10, where RuSH (LinkInvent) and LinkInvent shared 4,736 designs (10.3% of RuSH designs), and DeLinker and RuSH (REINVENT) shared 498 designs (20.8% of DeLinker designs).
- *Shape and pharmacophore similarity.* To quantify the design quality in terms of shape and pharmacophore similarity, we report two “shape” and “color” scores (eq 5) using both ROCS (as in the scoring function)^22^ and an RDKit implementation, as provided by DeLinker^12,50^ (to serve as external control). RuSH performed consistently better at generating designs with a high three-dimensional and pharmacophore similarity to the reference molecules (Table 1). The only exception is ADCY10, where DeLinker outperformed all approaches. In this context, RuSH’s full molecule generation in combination with our scoring function achieved consistently better results compared to link-only generation. When directly comparing Link-INVENT approaches, we observed comparable performance across 3D metrics, demonstrating Link-INVENT’s capability to generate high-performing designs when utilizing carefully selected 2D scoring criteria based on a reference molecule, in combination with molecular postprocessing. Here, unconstrained generation using RuSH exhibited superior performance relative to either Link-INVENT approach. For a comprehensive overview, we report the complete distributions for 3D scoring in Supporting Figure S8.

**Table 1:** Benchmarking Analysis^a^

target	method	designs (no.)	unique (%)	quality filter (no. and %)	scaffold div. (↑)	molecule dist. (↑)	scaffold dist. (↑)	RDKit shape and color (↑)	ROCS shape and color (↑)
PIM1	DeLinker	12,000	71%	5,597 (47%)	0.39	0.61 ± 0.06	**0.90** ± **.05**	0.63 ± 0.07	0.56 ± 0.07
	LinkInvent	109,956	100%	**101,756 (93%)**	0.32	0.62 ± 0.05	0.89 ± 0.04	0.59 ± 0.06	0.52 ± 0.07
	RuSH (LinkInvent)	108,547	100%	96,840 (89%)	**0.49**	**0.63** ± **.05**	0.89 ± 0.04	0.59 ± 0.07	0.52 ± 0.08
	**RuSH (REINVENT)**	6337	100%	^b^4,855 (77%)	0.38	0.46 ± 0.06	0.77 ± 0.06	**0.67** ± **.06**	**0.68** ± **.07**
	known scaffold-hop (**2**)	n.a.	n.a.	n.a.	n.a.	0.80	0.87	0.69	0.70
HIV1	DeLinker	13,500	3%	0 (00%)	-	-	-	-	-
	LinkInvent	116,994	100%	91 (00%)	0.25	0.84 ± 0.01	0.93 ± 0.01	0.43 ± 0.05	0.31 ± 0.04
	RuSH (LinkInvent)	2	100%	1 (50%)	-	-	-	-	-
	**RuSH (REINVENT)**	6237	100%	**2,294 (37%)**	**0.48**	**0.80** ± **.04**	**0.92** ± **.03**	**0.52** ± **.05**	**0.43** ± **.04**
	known scaffold-hop (**4**)	n.a.	n.a.	n.a.	n.a.	0.78	0.91	0.52	0.43
JNK3	DeLinker	12,000	68%	3,157 (26%)	0.35	0.58 ± 0.04	**0.93** ± **.06**	0.55 ± 0.05	0.44 ± 0.05
	LinkInvent	107,272	100%	55 970 (52%)	0.30	0.60 ± 0.02	0.91 ± 0.03	0.52 ± 0.05	0.41 ± 0.05
	RuSH (LinkInvent)	103,366	100%	50,232 (49%)	**0.57**	0.61 ± 0.03	0.89 ± 0.03	0.55 ± 0.05	0.44 ± 0.05
	**RuSH (REINVENT)**	9817	100%	^b^ **6,506 (66%)**	**0.57**	**0.62** ± **.06**	0.86 ± 0.05	**0.58** ± **.06**	**0.49** ± **.07**
	known scaffold-hop (**6**)	n.a.	n.a.	n.a.	n.a.	0.59	0.85	0.54	0.44
ADCY10	DeLinker	12,000	33%	2,891 (24%)	0.31	0.68 ± 0.07	0.90 ± 0.07	**0.80** ± **.08**	**0.72** ± **.08**
	LinkInvent	104,409	100%	45,002 (43%)	**0.53**	**0.70** ± **.04**	0.89 ± 0.04	0.68 ± 0.08	0.62 ± 0.07
	RuSH (LinkInvent)	103,893	100%	50,683 (49%)	0.46	**0.70** ± **.04**	0.90 ± 0.04	0.70 ± 0.08	0.63 ± 0.08
	**RuSH (REINVENT)**	10,388	100%	**9,950 (96%)**	^c^0.13	0.69 ± 0.06	**0.92** ± **.05**	0.76 ± 0.09	0.71 ± 0.09
	known scaffold-hop (**8**)	n.a.	n.a.	n.a.	n.a.	0.68	0.89	0.81	0.73

a Comparison of our approach with Link-INVENT, and DeLinker. As a control, our scoring function (SF) was also used in combination with LinkInvent. From left to right, the table reports metrics for (a) Number of (valid) designs and the fraction of unique designs, (b) design quality (fraction of unique designs that passed DeLinker quality filters), (c) scaffold diversity (Scaled Shannon Entropy [SSE]), molecular dissimilarity, and scaffold dissimilarity to the reference molecule, and (d) shape and pharmacophore similarity to the reference molecule (Shape and Color Scores) obtained by RDKit and ROCS. For similarity metrics, the average ± standard deviation is reported. The best value per target is highlighted in boldface. The (dis)­similarity of the know scaffold-hops compared to the reference is reported for comparison.

b A high number of designs were filtered out due to decoration variation (α = 0.9 for RL) (PIM1: 1468 (99%), JKN3: 3142 (94%) of filtered designs).

c Most scaffolds did not contain rings, which are considered the same Bemis–Murcko scaffold (“no scaffold”), leading to a reduced SSE.

These results show the potential of our approach to explore novel scaffold candidates, while at the same time preserving three-dimensional and pharmacophore information. Interestingly, our method remarkably outperforms all baselines on HIV1, which is the most challenging case in terms of structural complexity and number of decorations. This underscores the benefit of unconstrained generation and of “fuzzy” substructure matching in efficiently navigating the chemical space when multiple complex properties have to simultaneously be optimized.

## Conclusions and Outlook

This work introduced a new computational framework for scaffold hopping, via reinforcement learning with unconstrained molecular generation. RuSH was able to generate designs that retain a high three-dimensional similarity to a bioactive reference, while at the same time possessing novel scaffolds. Moreover, unconstrained generation allows for scaffold hopping with an arbitrary number of decorations, and to tune the desired structural variability in molecular decorations. Similarly, the ability to perform transfer learning proved particularly useful to increase the speed and efficiency of chemical space exploration, and to promote the matching of shape and pharmacophore properties. In principle, RuSH’s pipeline also allows for multiple references to be used, by combining the individual 2D and 3D scores via an aggregation function. Finally, the ScaffoldFinder algorithm can be adapted to virtually any type of substructure matching, e.g., generate designs with warheads, or coupling groups for improved synthetic accessibility.

For certain targets, one foreseen limitation of RuSH could be the difficulty of learning to include all decorations before score optimization can happen. For the considered cases, this was solved by transfer learning. Moreover, while we set out to use the well-established Tanimoto similarity on ECFPs, other similarity metrics may be more effective for scaffold hopping.^51^ Lastly, we acknowledge the impact and necessity of postprocessing in any pragmatic context for *de novo* drug design. As such, the quality of generated designs will depend as much on the postprocessing as it does on the generator in question. Future works may benefit from exploring the parameter space of the scoring function—in particular α and the aggregation weights *w*_s_, *w*_c_, *w*_2D_, *w*_3D_—for chemical space exploration and goal-directed optimization. Our findings suggest that, for each case study and design objective, a different set of scoring weights may be optimal. The balance between diversity, 2D distance and 3D similarity is unknown and will require large-scale experiments.

In light of recent developments in reinforcement learning frameworks,^10,30^ RuSH’s scoring function could be further combined with secondary objectives for multiobjective optimization and curriculum learning, e.g., to jointly improve the inclusion of decorations, considering several reference molecules simultaneously, and optimize for molecular properties like solubility or drug-likeness.

## Materials and Methods

### Reinforcement Learning Setup

#### Molecule Generation

Long–short-term memory (LSTM) networks^33^ were used as generators of SMILES strings. The LSTM Prior was trained on the GuacaMol data set,^20^ which contains 1,554,993 drug-like molecules curated from ChEMBL.^35^ All Prior models were trained used the REINVENT framework, using the default hyper-parameters of REINVENT.^52^ A single Prior model for the case studies was trained for 20 epochs, and used for all subsequent experiments. For the benchmark, additional data curation was performed for a fair literature comparison, following the “*REINVENT Community Data Preparation Notebook*”.^9^ The Prior models for the benchmark were trained for a total of 40 epochs, and models at epoch 20, 30, and 40 were selected and evaluated. Generated designs by these agents were aggregated for analysis. Fine-tuning was done by performing transfer learning on the SMILES string of reference molecules for a total of 20 epochs. 10,000 strings were sampled at each epoch, and used to select the fine-tuned model for reinforcement learning (as the one where the probability of sampling the reference molecule was above 5%, Supporting eq S2). Reinforcement learning was done for 2000 cycles for the case studies, and 1000 cycles for the benchmark. During reinforcement learning, 64 SMILES strings were sampled per cycle, using weighted random sampling with a temperature (*T*) of *T* = 1.

#### ScaffoldFinder Algorithm

We devised the ScaffoldFinder algorithm (Alg. 1) to determine the correct inclusion of the reference decorations in the generated designs. ScaffoldFinder takes the following inputs: (a) one (or more) molecule(s), (b) a set of reference decorations, and (c) an allowance parameter (**α**), determining the “fuzzyness” of the substructure matching. Given one molecule and one decoration, ScaffoldFinder proceeds as follows:

(1) “*Fuzzy’*”*substructure matching*. The algorithm identifies the largest common structure (maximum common substructure, MCS) between the decoration and the molecule it is currently evaluating. This is achieved via the “Find Maximum Common Substructure” (FMCS) algorithm.^27^ The exact matching performed by FMCS is extended by allowing for “dummy atoms” to match any nonperipheral heavy atom, and ensuring that such atoms have not been included in previous decorations. To this extent, the algorithm remembers a list of previously identified atoms. The algorithm will only accept a match if (a) the dummy atom is part of the MCS, (b) connected by a single bond, and (c) is matched with an atom that has a degree greater than 1. Additionally, the size of the match (in terms of the number of atoms) must be at least α times the size of the decoration.
(2) *Selection of the largest common substructure*. If the accepted MCS matches with more than one possible substructure in the molecule (Figure 2b, top decoration), the best match is chosen by maximizing the number of atoms on the other size of the matched dummy atom. In other words, we choose the MCS that is located “furthest away”, or most peripheral in the molecule.
(3) *Cleavage*. As a final step, the algorithm will perform a test cleave by segmenting the molecule at the bond between the decoration and the dummy atom. If the molecule can be cleaved in two with chemical validity, it proceeds. The algorithm then perform a final check for the size of the cleaved decoration, and compares it to the original size of the decoration. For the match to be valid, the number of atoms in the cleaved decoration must not differ from the decoration’s size by more than a factor of α.

If this process succeeded, the atoms of the cleaved decoration are stored, and the process repeats for the remaining input decorations. This process can be loosely formulated as a Success score (*S*_s_), determined as follows

1 $$S_{s}=\frac{\sum_{r}^{R}\delta \left(r\right)}{\left|R\right|},\mathrm{with},\delta \left(r\right)=\{\begin{array}{l} 1,\mathrm{if}\;d_{r}\;\mathrm{and}\;m_{r}<n_{d_{r}}<M_{r}\\ 0,\mathrm{otherwise} \end{array}$$

where *r* runs over the set of total reference decorations (*R*). For any reference decoration *r*, the variables *d*_*r*_ and *n*_*d*_*r*__ represent the corresponding cleaved decoration (if it is found), and the number of atoms in it. The variables *m*_*r*_ and *M*_*r*_ represent the minimum and maximum size allowed for the cleaved decoration, and depend on *r*. These values are controlled by a user-defined parameter α as follows

2 $$\begin{array}{l} m_{r}=n_{r}\cdot \alpha \\ M_{r}=n_{r}\cdot \alpha^{-1} \end{array}$$

where *n*_*r*_ is the number of atoms in the reference decoration *r*. For example, if α is 0.8, and the reference decoration contains 10 atoms (Figure 2a), cleaved decorations with between 8 and 12 atoms are permitted (Figure 2b, top decoration). This implementation allows one to leverage unconstrained generation further, by permitting exploration of the decorations in addition to the scaffold. This furthermore improves agent learning of decoration inclusion by permitting small errors in substructure matching.

Finally, If all decorations pass this process (*S*_s_ = 1.0), the identified decorations are cleaved, dummy atoms are added to the scaffold, and the scaffold is returned. In all cases, the success score (*S*_s_) is returned for evaluation and (partial) scoring. ScaffoldFinder works for any number of decorations that are connected by single bonds to a central scaffold. In this work, the decorations have been determined based on literature definitions, i.e., the substructures shared between the reference and ground truth molecules (Figure 3). Throughout this work, a constant value of α = 0.9 was used. This was chosen to demonstrate a range of decoration variation across the four case studies. This way, no variation was permitted for the smallest decoration. For benzene derivatives one atom variation was permitted. For the largest decorations up to two atoms.

#### Design Scoring

Designs were evaluated according to the following steps.

##### Molecule Filter

The generated designs are filtered according to their molecular weight, number of rotatable bonds, and number of stereocenters. These properties were calculated for the reference molecules, and thresholds for filtering were chosen to be reasonably above those that were calculated (Supporting Table S1). Molecules filtered this way are not rewarded.

##### Identification of Scaffolds and Decorations

A set of scaffolds and terminal decorations is obtained via ScaffoldFinder algorithm (Alg. 1). Molecules containing only part of the decorations of the reference molecule are given a partial reward of 0.3, and are not scored further. The remaining molecules proceed to the 2D and 3D scoring.

##### 2D Reward

2D reward (*S*_2D_) is computed by comparing the scaffold of the design to the scaffold of the reference molecule, as obtained by ScaffoldFinder. This is achieved by computing extended connectivity fingerprints^29^ (ECFP; radius = 3, 2048 bits) of both scaffolds, and calculating the pairwise dissimilarity as Jaccard distance^23^

3 $$S_{2D}\left(\mathrm{Mo}l_{i},\mathrm{Mo}l_{ref}\right)=1-\frac{\left|V_{i}\cap V_{ref}\right|}{\left|V_{i}\cup V_{ref}\right|}$$

where *V*_*i*_ and *V*_ref_ denote the sparse bit-vectors (ECFPs) of the scaffolds of the *i*th design and of the reference, respectively. *S*_2D_ ranges from 0 to 1. The greater the score, the less similar two scaffolds are in terms of their substructures.

##### 3D Reward

The 3D reward is computed by generating a geometry-optimized conformer ensemble of up to 32 conformers for each enumerated isomer of each succeeded molecule using OMEGA.^31^ 32 conformers were chosen as a good trade-off between runtime and ROCS scoring in preliminary analyses (see Supporting Figure S9). Macro-cycles are filtered by OMEGA and were not considered in this work.^31^ The ensembles are then aligned and scored against the bioactive conformation of the reference molecule(s) by ROCS.^22^ ROCS provides two similarity scores (0.0–1.0) for every pair of conformations, based on how well their shapes (score *S*_S_, “shape”) and pharmacophore features (score *S*_C_, “color”) of the two molecules overlap (the higher the scores, the better the overlap). These scores are used to compute the reward for each conformer in the ensemble as a weighted mean, as follows

4 $$\rho \left(C_{\mathrm{ij}},C_{\mathrm{ref}}\right)=\frac{S_{S}\left(C_{\mathrm{ij}},C_{\mathrm{ref}}\right)\cdot w_{s}+S_{C}\left(C_{\mathrm{ij}},C_{\mathrm{ref}}\right)\cdot w_{c}}{w_{s}+w_{c}}$$

where *C*_*ij*_ is the *j*th conformer of the *i*th design, and *Mol*_ref_ is the reference molecule in bioactive conformation; *S*_S_ and *S*_C_ are the ROCS scores obtained by comparing the chosen conformations, and *w*_s_ and *w*_c_ their respective weights. In this work, constant at *w*_s_ = 1.0 and *w*_c_ = 1.2. Equation 5 allows one to tune the contribution of shape and pharmacophore features in the final score. These differential weights are based on preliminary results, which showed that the ROCS Shape score was easier to satisfy as a criterion. ROCS Color score tended to be numerically lower. Finally, each design is given the 3D reward corresponding to that of the highest scoring conformer

5 $$S_{3D}\left(\mathrm{Mo}l_{i},\mathrm{Mo}l_{\mathrm{ref}}\right)=\max_{C_{\mathrm{ij}}\in \mathrm{Mo}l_{i}}\rho \left(C_{\mathrm{ij}},C_{\mathrm{ref}}\right)$$

##### Final Reward

Finally, every *i*th design is given a final reward (*S*_F_), computed as the weighted harmonic mean between the 2D and 3D scores, as

6 $$S_{F}\left(\mathrm{Mo}l_{i},\mathrm{Mo}l_{\mathrm{ref}}\right)=\frac{w_{2D}+w_{3D}}{w_{2D}\cdot S_{2D}^{-1}+w_{3D}\cdot S_{3D}^{-1}}$$

where *S*_2D_ and *S*_3D_ are the 2D and 3D scores, and *w*_2D_ and *w*_3D_ are the associated weights. These weights were set constant at *w*_2D_ = 1.5 and *w*_3D_ = 1.0. Weights were chosen heuristically to align with literature findings on Tanimoto (dis)­similarity scoring using ECFPs.^47,53^ Furthermore, preliminary results showed need to emphasize 2D distance when jointly optimizing for 3D similarity, while preserving diversity. Equation 6 ensures that optimizing for one reward does not come at the expense of the other. Mentioned weights are intended to be tunable by the user, and may be further explored in future work.

#### Reinforcement Learning Loss

To ensure generated SMILES strings remain “chemically plausible”,^9^ the average negative log-likelihood (*NLL*) of every generated string is calculated using the Prior model (*NLL*_Prior_). This serves as a measurement of how well the generated SMILES string aligns with the learned distribution of ChEMBL. This *NLL*_Prior_ is then combined with the scoring function reward, to form an “augmented negative log-likelihood” (*NLL*_Aug_),^37,54^ defined as

7 $$\mathrm{NL}L_{\mathrm{Aug}}\left(i\right)=\mathrm{NL}L_{\mathrm{Prior}}\left(i\right)-\sigma \cdot S_{F}\left(i\right)$$

where *NLL*_Aug_(*i*) is the augmented NLL for the *i*th SMILES string, and *S*_F_(*i*) the final scoring function reward (eq 6). This *NLL*_Aug_ will serve as the target distribution for the agent to tend toward. The (user-defined) σ factor balances reward and likelihood, and was kept constant at σ = 128 ^52^.

Finally, the *NLL*_Aug_ is used to update the policy of the agent, via the following loss^37^

$$\mathrm{loss}=\frac{1}{N}\overset{N}{\underset{i=1}{\sum }}(\mathrm{NL}L_{\mathrm{Aug}}\left(i\right)-{\mathrm{NLL}}_{\mathrm{Agent}}(i){)}^{2}$$ 8

where *i* runs over the *N* generated SMILES, and *NLL*_Aug_ and *NLL*_Agent_ are the augmented log-likelihood and the agent log-likelihood, respectively. In other words, the loss captures how well the agent’s current output SMILES align with the ideal outputs (represented by *NLL*_Aug_). By minimizing the loss via stochastic gradient descent, the agent progressively adjusts its policy to generate SMILES strings that are both chemically plausible (minimizing *NLL*_Prior_) and desirable according to the scoring function (maximizing *S*_F_).

### Scaffold Hopping Experiments

#### Target and Reference Selection

Structures **1** ^38^ (PDB-ID: 5DWR), **3** ^46^ (PDB-ID: 1AJV), **5** ^40^ (PDB-ID: 3FI3), and **7** ^41^ (PDB-ID: 5IV4) were used the starting point (reference) for scaffold hopping. Known scaffold hops were used to evaluate the reinforcement learning success, i.e., **2** ^38^ (PDB-ID: 5KZI), **4** ^55^ (Docked and computationally validated by Bergmann and co-authors^39^), **6** ^40^ (PDB-ID: 3FI2), and **8** ^41^ (PDB-ID: 8CO7).

#### Recovery of Known Scaffold-Hops

For each target, we computed *t*-distributed stochastic neighbor embedding (t-SNE) (number components = 2, perplexity = 100 perplexity, random seed = 42)^42^ on the ECFP6s^56^ of the *de novo* designs. From the t-SNE coordinates, we retrieved the designs that fell within a ± 2 coordinate window around the published hops (**2**, **4**, **6**, and **8**). The designs were then ranked using the scoring function award. We then visually inspected the 16 highest scoring designs for structural similarity to the reference structures and further discussion (Supporting Figures S3–S6).

#### Advanced Post-Processing

For our in-depth design analysis, we filtered designs for (predicted) properties with relevance for *de novo* design. This was achieved partially by Novartis in-house models, to predict (a) Permeability (low-efflux [LE],^57^ and parallel artificial membrane [PAMPA][Bibr ref58]), (b) human clearance, (d) octanol–water partitioning coefficient (log *P*), (e) Severity Score^59^ for reactivity, toxicity and stability, and (f) “chemical intuition” and desirability of the structures.^60^ The thresholds were based on Novartis expertise, Lipinski’s rule of five,^61^ and data-driven considerations (Supporting Table S9). The molecules that were retained were subjected to molecular docking.

#### Molecular Docking

Protein structures of the known scaffold hops were downloaded from the PDB, and prepared using Schrödinger Maestro^62^ (default settings). For each target, the designs were prepared using Schrödinger LigPrep,^63^ docked using Glide,^43^ and scored with Hyde.^44,64^ References molecules were subjected to the same pipeline as the generated designs (self-docking), and can be found in Supporting Table S11. Designs that could successfully be docked were filtered for internal energy, docking score, torsion quality and intramolecular clash (Supporting Tables S12). The remaining designs were then manually inspected for relevant interactions in the crystal structure of the reference molecules. The docking and scoring statistics for the selected designs can be found in Supporting Table S8.

### Benchmarking

#### Literature Methods

The literature methods were used as follows:

- DeLinker was used as in the original repository.^65^ Their “fragment linking” approach was applied using the reference molecules’ decorations as input. Generation parameters were adjusted for each reference molecule. We generated scaffolds that varied by ±4 atoms in size from the reference scaffold (5–12 PIM1, 20–28 HIV1, 5–12 JNK3, 3–10 ADCY10). 1500 designs were sampled per scaffold size for a total sample size of 12000 (13500 for HIV1) per ligand.
- Link-INVENT was used as in the original repository.^66^ In agreement with the original paper,^15^ we tuned the scoring functions for each case target based on the binding mode and structural properties of the reference molecule. The properties molecular weight, number of hydrogen bond donors, number of hydrogen bond acceptors, and number of rings were considered as components for the scoring functions. All scores were transformed using (double) sigmoid (*k* = 0.15, div = 500, se = 250, si = 250), and can be found in Supporting Table S13. For example, designs were rewarded for having a molecular weight of ±60 g/mol compared to the reference molecule. REINVENT default reinforcement learning hyper-parameters were used^52^ to match those of RuSH, batch-size was 128. Reinforcement learning was performed for 1000 epochs.

#### Scaffold Diversity

Scaffold diversity was computed based on a previously published approach,^49^ by obtaining the cyclic substructures of the generated scaffolds, using the Bemis–Murcko algorithm.^36,50^ We report the Scaled Shannon Entropy (SSE) to quantify the scaffold diversity within a set, with SSE = 0 indicating that all scaffolds are the same, and SSE = 1 indicating that all scaffolds are different. For a comprehensive explanation, we refer to Supporting eq S1.

#### Generative Ability

To fairly estimate the quality of generated designs, we followed the postprocessing workflow of DeLinker.^12^ We report the number of molecules that passed the following evaluations: Molecules were evaluated for the exact fragment inclusion (i.e., the input decorations for the linker-based methods. For HIV1, the methoxyphenyl decorations were used), the presence of Pan-Assay Interference Compounds (PAINS),^67^ synthetic accessibility,^68^ and ring aromaticity. For a complete estimation, we introduced an additional check whether all input decorations were present correctly (using ScaffoldFinder, α = 1). For our approach, reinforcement learning resulted in nonexact fragments, which were removed by DeLinker postprocessing (PIM1:1468 designs, 99%; JKN3:3142 designs, 95%).

#### Shape and Color Matching

ROCS scoring was implemented as previously explained (eq 5). RDKit Shape and Color Scoring was implemented as in recent works,^12,15^ obtained from the DeLinker repository.^65^

### Software and Code

All RuSH experiments were performed using the REINVENT v3.2 framework and environment.^9^ ECFPs (no. bits = 2048, radius = 3), Bemis–Murcko scaffolds, and molecular descriptors were computed with RDKit^50^ v 2020.3.3.0. For the extended FMCS algorithm, RDKIT version 2023.3 or greater is required. Scikit-learn version 0.21.3 was used for t-SNE dimensionality reduction. OpenEye licensed software OMEGA version 4.1.0.2 and ROCS version 3.4.1.2 were used. Wherever applicable, the randomization was seeded with 42.

## Supplementary Material



## Data Availability

The code to reproduce transfer learning, reinforcement learning, and baseline experiments is available at https://github.com/molML/RUSH with example notebooks for PIM1. The code for REINVENT and Link-INVENT is available at https://github.com/MolecularAI/Reinvent. The code for DeLinker is available at https://github.com/oxpig/DeLinker. For the use of RUSH, an OpenEye software license is required. We also provide a standalone script for using ScaffoldFinder, which is demonstrated in using_scaffoldfinder.ipynb.

## Notes

Additional experimental details, methods, and results, including the effect of α on reinforcement learning (PDF)
