# Supplementary material for: Scaffold Hopping with Generative Reinforcement Learning
Source: J Chem Inf Model. 2025 Jun 26;65(13):6513–25. doi: 10.1021/acs.jcim.5c00029 (PMC12264953; doi:10.1021/acs.jcim.5c00029)
Supplement: Supplementary file 1 [file ci5c00029_si_001.pdf]

SUPPORTING INFORMATION

Scaffold Hopping with Generative Reinforcement Learning

L. Rossen, F. Sirockin, N. Schneider, F. Grisoni

CONTENTS

I ScaffoldFinder Algorithm 2

II Reinforcement Learning Case Studies 3
- II-A Molecule filtering prior to scoring . . . 3
- II-B Reinforcement Learning Behaviour . . . 3
- II-C Scaled Shannon Entropy for Scaffold Diversity . . . 4
- II-D Selection of fine-tuning epochs . . . 4
- II-E Effect of transfer learning on reinforcement learning . . . 5
- II-F Recovery of Known Scaffold-Hops . . . 6
  - II-F1 HIV1 TL . . . 6
  - II-F2 HIV1 . . . 7
  - II-F3 JNK3 TL . . . 8
  - II-F4 ADCY10 TL . . . 9
- II-G Discovery of New Scaffold-Hops . . . 10
- II-H Molecular Property Filters . . . 11
- II-I Molecular Docking . . . 11

III Benchmarking 12
- III-A LinkInvent . . . 12

IV Conformer Generation 13

V Effect of allowance (α) on reinforcement learning 14

I. ScaffoldFinder Algorithm

A pseudo-code for the `ScaffoldFinder` algorithm is provided below. Given a list of molecules, decorations (to keep), and an allowance factor (α), it will iteratively perform substructure matching on the molecules, using the decorations. If all decorations were successfully identified, which depends on α, the decorations are cleaved and the remaining structures are returned as scaffolds. In practice, multiple sets of decorations can be passed as a nested list (if more than one reference molecule is available). In this case, the algorithm returns the first scaffold found, if any.

Algorithm 1 ScaffoldFinder.

```
Input: molecules, decorations, allowance
Output: success, scaffolds
  M, D, α ← molecules, decorations, allowance
  for mol in M do
      I_d' ← [ ]                                   ▷ previously identified decoration indices
      success ← 0.0
      for d in D do
          MCS ← FMSC(mol, d, I_d')                 ▷ [27]
          B ← BestMatch(mol, MCS, I_d')
          d' ← Cleave(mol, B)
          if d' and α|d| < |d'| < |d|/α then
              I_d' ← +i_d'
              success ← +1/|D|
          end if
      end for
      if success is 1 then                         ▷ all decorations matched
          scaffold ← Cleave(mol, I_d')
          scaffolds ← +scaffold
      end if
  end for
```

II. Reinforcement Learning Case Studies

A. Molecule filtering prior to scoring

This section contains the parameters used for each case study to filter out (penalize) designs by basic molecular properties. The molecules were filtered by the number of stereocenters, the molecular weight, and the number of rotatable bonds. These filters serve as a computationally cheap filter to ensure that agents reach and stay within a desirable chemical space close to that of the reference molecule(s).

TABLE S1: Thresholds for the molecular property filters. Properties were calculated for each reference ligand, and thresholds were set at a rounded margin of +20% above those. The purpose of these filters is to minimize computational redundancy of 3D scoring. We assume molecules above these thresholds are too large/complex in comparison to the reference molecule to fall within chemical space of interest.

Target	stereocenters	mol. weight	rot. bonds
PIM1	6	500	10
HIV1	5	630	12
JNK3	4	600	10
ADCY10	4	350	8

B. Reinforcement Learning Behaviour

Comprehensive results of the learning behavior experiments. The experiments are color-coded according to their respective case study. Furthermore, computational run-time is plotted in addition to the batch-average scores, to provide further insight into agent learning behavior and reward profiles.

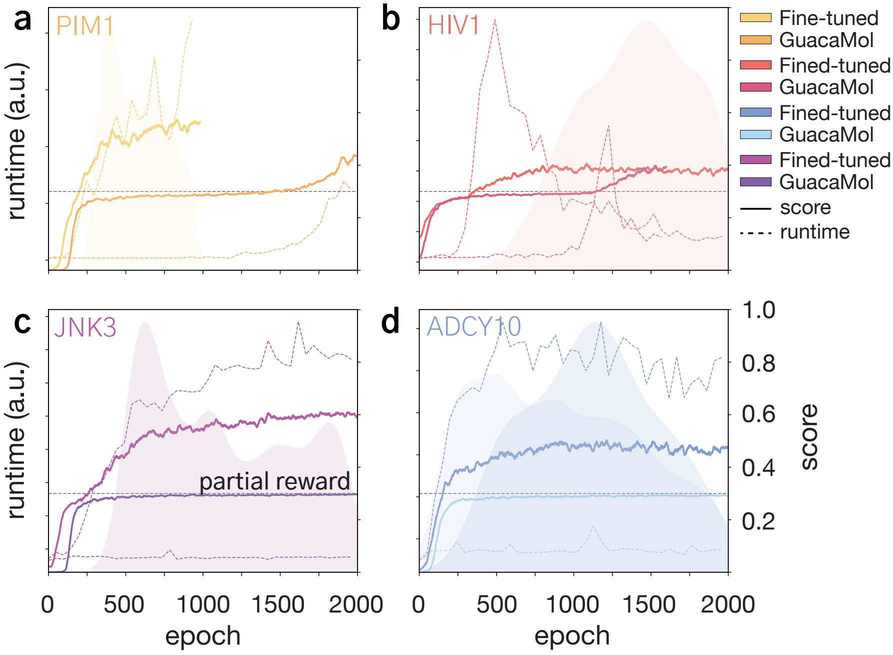

Fig. S1: **Reinforcement learning experiments for the chosen protein targets. (a)** PIM1, **(b)** HIV1, **(c)** JNK3, and **(d)** ADCY10. For each target we compare the results of the ChEMBL Prior with a fine-tuned Prior (using the reference molecule). Depicted is the average score awarded by the scoring function (64 SMILES sampled per epoch), together with the qualitative compute time (a.u.) per epoch. Additionally shown are the normalized distribution plots for generated designs that scored higher than 0.7 (and 0.9, darker shade for ADCY10) to illustrate when high scoring designs were generated.

C. Scaled Shannon Entropy for Scaffold Diversity

The scaled Shannon entropy (SSE) for the measurement of scaffold diversity is defined as [49]:

$$SSE_n = \frac{\sum_{i=1}^{n} p_i \cdot log_2(p_i)}{log_2(n)} \; ; \; p_i = c_i/P \tag{S1}$$

Where p_i is the relative frequency of a Bemis-Murcko cyclic substructure (BM) in a population of P compounds; n the number of most-common BM to consider, and c_i the absolute frequency (count) of scaffolds that contain the BM at index i. The values of SSE range between 0, where all scaffolds share the same BM, and 1.0, where each scaffold has a unique BM. SSE values closer to 1.0 indicate large scaffold diversity within the n most populated BM. When not specified, we compute the SSE over the entire set of BM, *i.e.*, n = *'all'*.

This relative frequency as a function of BM can also be plotted, termed 'cyclic-retrieval curve', or CSR. In order to compare the CSR quantitatively, we report the fraction of BM required to retrieve 50% (F_{50}) of the scaffold population. I.e., F_{50} denotes the fraction of unique cyclic substructures found in 50% of the generated scaffolds. F_{50} ranges for 0.0 to 0.5 where the CSR curve is a straight line at $y = x$. Thus, a larger F_{50} value indicates higher scaffold diversity.

TABLE S2: Diversity metrics F_{50} & Scaled Shannon Entropy (SSE) for the Bemis-Murcko [36] (BM) scaffold diversity analysis [49]. F_{50} scores are derived from retrieval curves and denote the fraction of unique BMs contained in 50% of the population (Fig. 5, left). SSE was computed on the total distribution of populated scaffolds (Fig. 5, right). For both metrics, higher values indicate higher diversity.

	**PIM1 Guac.**	**PIM1 TL**	**HIV1 Guac.**	**HIV1 TL**	**JNK3 TL**	**ADCY10 TL**
F_{50}	0.05	0.02	0.19	0.19	0.01	0.01
SSE_{all}	0.55	0.63	0.94	0.95	0.48	0.48

D. Selection of fine-tuning epochs

The fine-tuning epoch was selected as the one where the reference molecule was sampled at a rate above p_{sample} in a given batch of molecules, as follows:

$$p_{sample} = 1 - \sqrt[batch]{1 - P_{sample}} \tag{S2}$$

Where P_{sample} is the probability of the exact reference molecule being sampled (in this work chosen to $P_{sample} = 0.05$. In other words, we fine-tuned the Prior until such that it has a 5% chance of generating the reference molecule in a batch 64 molecules. $p_{sample} = 1 - \sqrt[64]{0.95} = 8 \times 10^{-4}$.

E. Effect of transfer learning on reinforcement learning

To better understand the effect of TL on the learning behaviour and generative diversity of a RL agent, we additionally performed shorter RL runs for the other 14 fine-tuned JNK3 Priors. We chose JNK3 specifically because of the difficulty to sample the reference molecule during TL and possible over-biasing as a consequence. These results are shown in Supplementary Fig. S2. For the first 7 TL agents we observe the same behaviour as for the ChEMBL Prior (Supplementary Fig. S2 a). From TL agent 8 we observed agents that generate designs that satisfy the SF, and observe that for each extra epoch of TL, the agent learned to include both decorations earlier. We observed similar score convergences for all of these agents, at ca. 0.6. We observed no impactful change in diversity metrics as a function of transfer learning (Supplementary Fig. S2 b-c). Overall, agents shared between 50 - 80% of the most common scaffolds between all of them, and commonality increased as a function of TL (Supplementary Table S3).

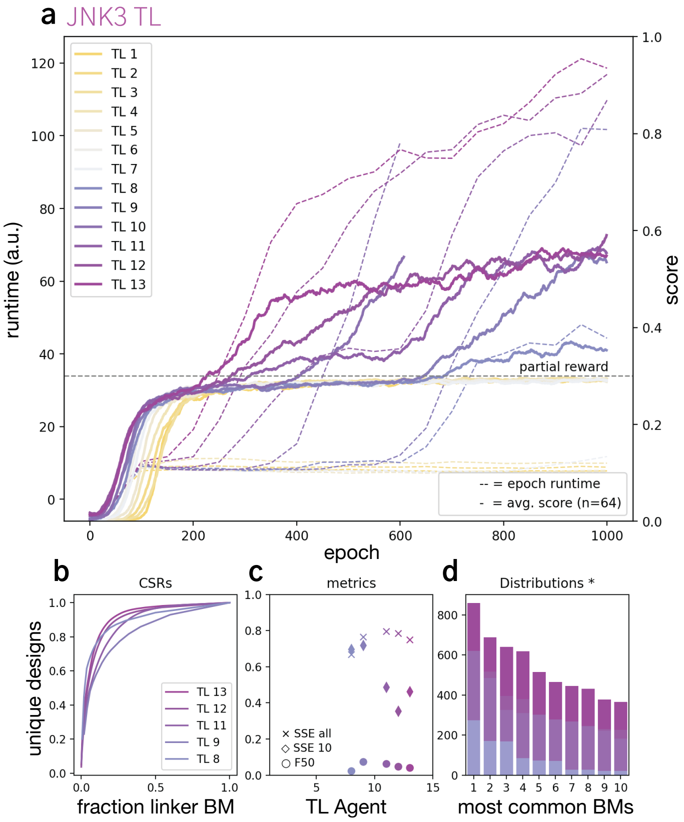

Fig. S2: **Effect of transfer learning on reinforcement learning performance**. (**a**) Reinforcement learning runs for JNK3 for all fine-tuned agents (1 through 14 epochs of transfer learning ('TL').), (**b-d**) scaffold diversity analysis [49] for runs that produced designs that scored above 0.4 (bottom panels): (**b**) cyclic retrieval curves, (**c**) diversity metrics as a function of transfer learning: Scaled Shannon Entropy (SSE) computed for the total distribution (all), and most common Bemis-Murcko (BM) cyclic substructures [36] (top 10), together with the fraction of cyclic substructures contained in 50% of the generated designs (F50) [49], (**d**) the frequency of the 10 most common BM scaffolds. *Different scaffolds per agent, see Supplementary Table S3.

TABLE S3: F_{50} & Scaled Shannon Entropy (SSE) of the generated scaffolds, together with the SMILES of the top 10 most generated BM scaffolds for the JNK3 TL agents that produced designs above 0.4. Missing SMILES refers to flexible scaffolds (no BM scaffold).

**JNK3**	**TL 8**	**TL 9**	**TL 10**	**TL 11**	**TL 12**	**TL 13**	**TL 14**
F_{50}	0.02	0.09	0.06	0.06	0.05	0.04	0.06
SSE_{all}	0.67	0.83	0.83	0.79	0.79	0.75	0.79
1	c1ccncc1	c1ccccc1	c1ccc2cnccc2c1	c1ccncc1	c1cncnc1	c1ccc2ncccc2c1	c1ccccc1
2	c1ccccc1	c1ccc2cnccc2c1	c1ccccc1	c1ccccc1	c1ccncc1	c1ccncc1	c1ccncc1
3	c1cncnc1	c1ccc2ncccc2c1	c1cn[nH]c1	c1ccc2oncc2c1	c1ccc2ncncc2c1	c1ccc2ncncc2c1	c1ccc2ncccc2c1
4		c1ccc2[nH]ccc2c1	c1ccncc1	c1cncnc1	c1ccc2ncccc2c1	c1ccc2cnccc2c1	c1ccc2ncncc2c1
5	c1ccsc1	c1ccncc1	c1ccc2ncncc2c1	c1ccc2ncccc2c1	c1ccc2cnccc2c1	c1cncnc1	c1cncnc1
6	c1cscn1	c1ccc2ncncc2c1	c1ccc2ncccc2c1	c1ccc2occc2c1	c1cn[nH]c1	c1ccc2[nH]ncc2c1	c1ccc2cnccc2c1
7	c1cn[nH]c1	c1cc[nH]c1	c1cncnc1	c1ccc2cnccc2c1	c1ccc2oncc2c1	c1ccccc1	c1ccc2[nH]ncc2c1
8	c1c[nH]cn1	c1ccc2occc2c1	c1ccc2[nH]cnc2c1	c1ccc2sccc2c1	c1ccccc1	c1ccc2[nH]ccc2c1	c1ccc2[nH]ccc2c1
9	c1ccnnc1	c1ccc2oncc2c1	c1ccc2oncc2c1	c1ccc2[nH]ccc2c1	c1ccc2[nH]ncc2c1	c1ccc2ccccc2c1	c1ccc2oncc2c1
10	c1ccc2ncncc2c1	c1ccc2[nH]cnc2c1	c1ccc2[nH]ncc2c1	c1ccc2[nH]ncc2c1	c1ccc2[nH]ccc2c1	c1ccc2sccc2c1	c1cnc2cnccc2c1

F. Recovery of Known Scaffold-Hops

This section shows the structures of the top scoring designs in close proximity to the published literature scaffold hop (ground truth). For each case, the most similar structure is highlighted with a yellow circle. Post-processing statistics are shown in the tables below, with the highlighted structure in **bold**.

1) HIV1 TL

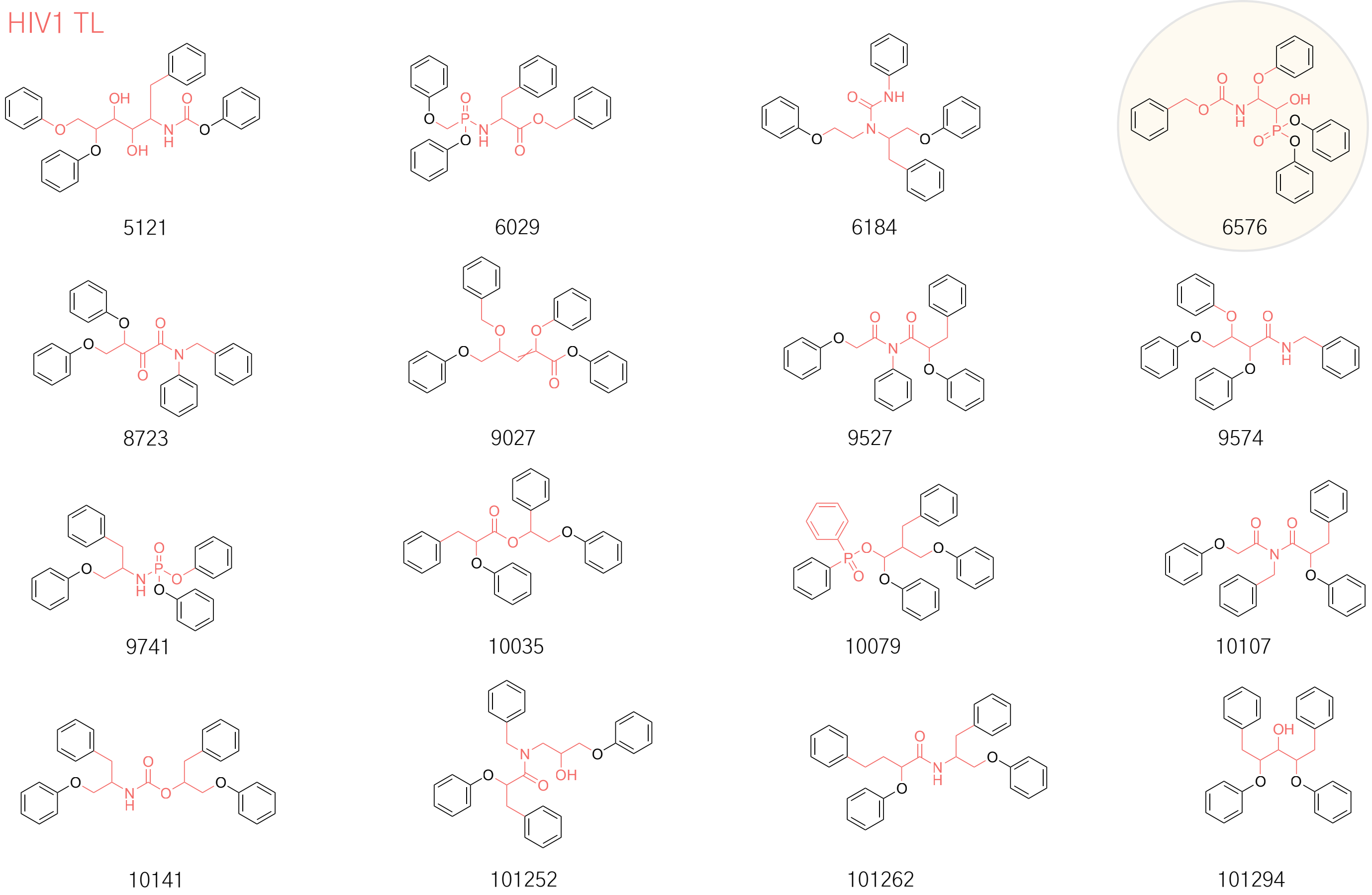

Fig. S3: 16 Highest scoring HIV1 TL designs around the published hop H2. Highlighted in yellow is the most similar structure.

TABLE S4: 16 Highest scoring HIV1 TL designs around the published hop. *Predicted values. Clearance, permeability and LogP models are Novartis global models trained on in-house data.

**ID**	**score**	**epoch**	**clear. Human* [uL/min/mg]**	**perm. LE* [cm-6/s]**	**perm. PAMPA* [cm/s]**	**LogP***	**Severity Score*** [59]	**MolSkill*** [60]
5121	0.61	641	>300	>5	$>$ -4.5	4.2	10	12.7
6029	0.6	357	inconcl.	>5	$>$ -4.5	4.6	0	18.9
6184	0.6	455	>300	inconcl.	inconcl.	5.0	0	1.5
**6576**	**0.6**	**435**	**inconcl.**	$>$ **5**	$>$ **-4.5**	**3.9**	**0**	**36.0**
8723	0.58	483	>300	>5	inconcl.	4.3	0	2.7
9027	0.57	536	inconcl.	>5	inconcl.	4.4	1	31.7
9527	0.57	577	>300	>5	inconcl.	4.8	0	3.9
9574	0.57	552	>300	>5	inconcl.	4.5	0	9.8
9741	0.57	396	>300	>5	$>$ -4.5	4.3	10	23.3
10035	0.56	409	>300	inconcl.	inconcl.	4.8	0	10.3
10079	0.56	421	inconcl.	≤ 1.5	$\leq$ -5.3	4.9	1	26.3
10107	0.56	601	>300	>5	inconcl.	4.5	0	6.8
10141	0.56	628	inconcl.	inconcl.	inconcl.	5.2	0	7.0
10252	0.56	630	>300	inconcl.	inconcl.	4.4	0	8.5
10262	0.56	1539	>300	>5	inconcl.	4.8	0	11.6
10294	0.56	558	>300	inconcl.	inconcl.	4.7	0	19.1

2) HIV1

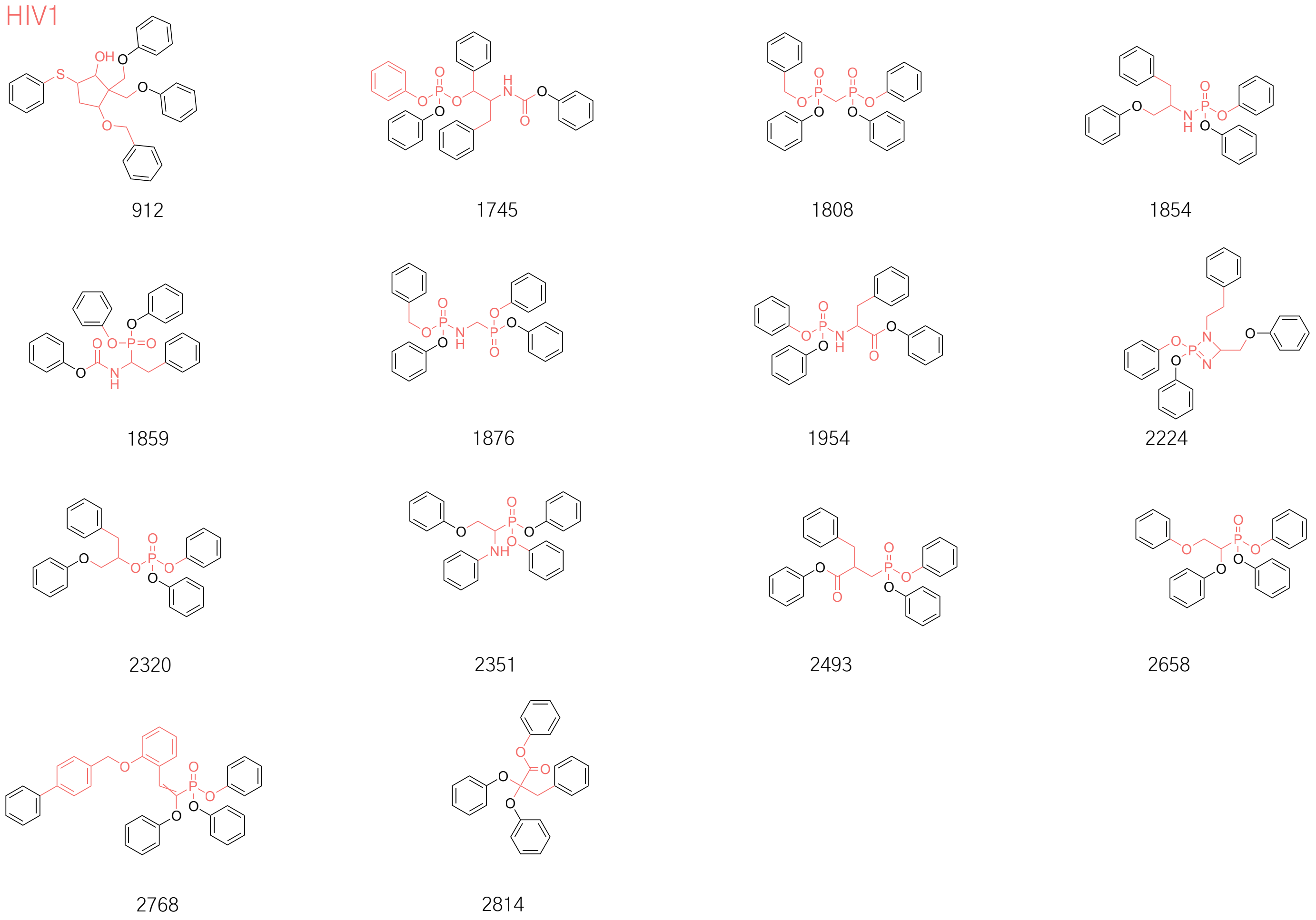

Fig. S4: 14 Highest scoring HIV1 designs around the published hop H2.

TABLE S5: 14 Highest scoring HIV1 designs around the published hop. *Predicted values. Clearance, permeability and LogP models are Novartis global models trained on in-house data.

ID	score	epoch	clear. Human* [uL/min/mg]	perm. LE* [cm-6/s]	perm. PAMPA* [cm/s]	LogP*	Severity Score* [59]	MolSkill* [60]
912	0.61	1394	> 300	> 5	inconcl.	5.0	0	18.5
1745	0.57	714	> 300	> 5	inconcl.	4.4	0	21.6
1808	0.57	876	> 300	inconcl.	inconcl.	3.9	10	37.5
1854	0.57	1326	> 300	> 5	> -4.5	4.3	10	23.3
1859	0.57	1221	inconcl.	> 5	> -4.5	4.2	0	21.9
1876	0.57	1534	> 300	> 5	> -4.5	2.7	10	37.4
1954	0.56	1381	> 300	> 5	> -4.5	4.0	10	18.9
2224	0.56	1342	inconcl.	inconcl.	inconcl.	4.8	1	20.0
2320	0.55	1596	inconcl.	inconcl.	inconcl.	4.4	0	19.3
2351	0.55	1377	inconcl.	> 5	inconcl.	4.6	0	19.2
2493	0.55	1577	inconcl.	inconcl.	inconcl.	4.2	0	22.5
2658	0.54	1377	inconcl.	inconcl.	inconcl.	4.7	0	31.6
2768	0.53	1265	≤ 100	≤ 1.5	≤ -5.3	5.1	1	37.0
2814	0.53	1308	inconcl.	> 5	inconcl.	5.0	0	18.0

3) *JNK3 TL*

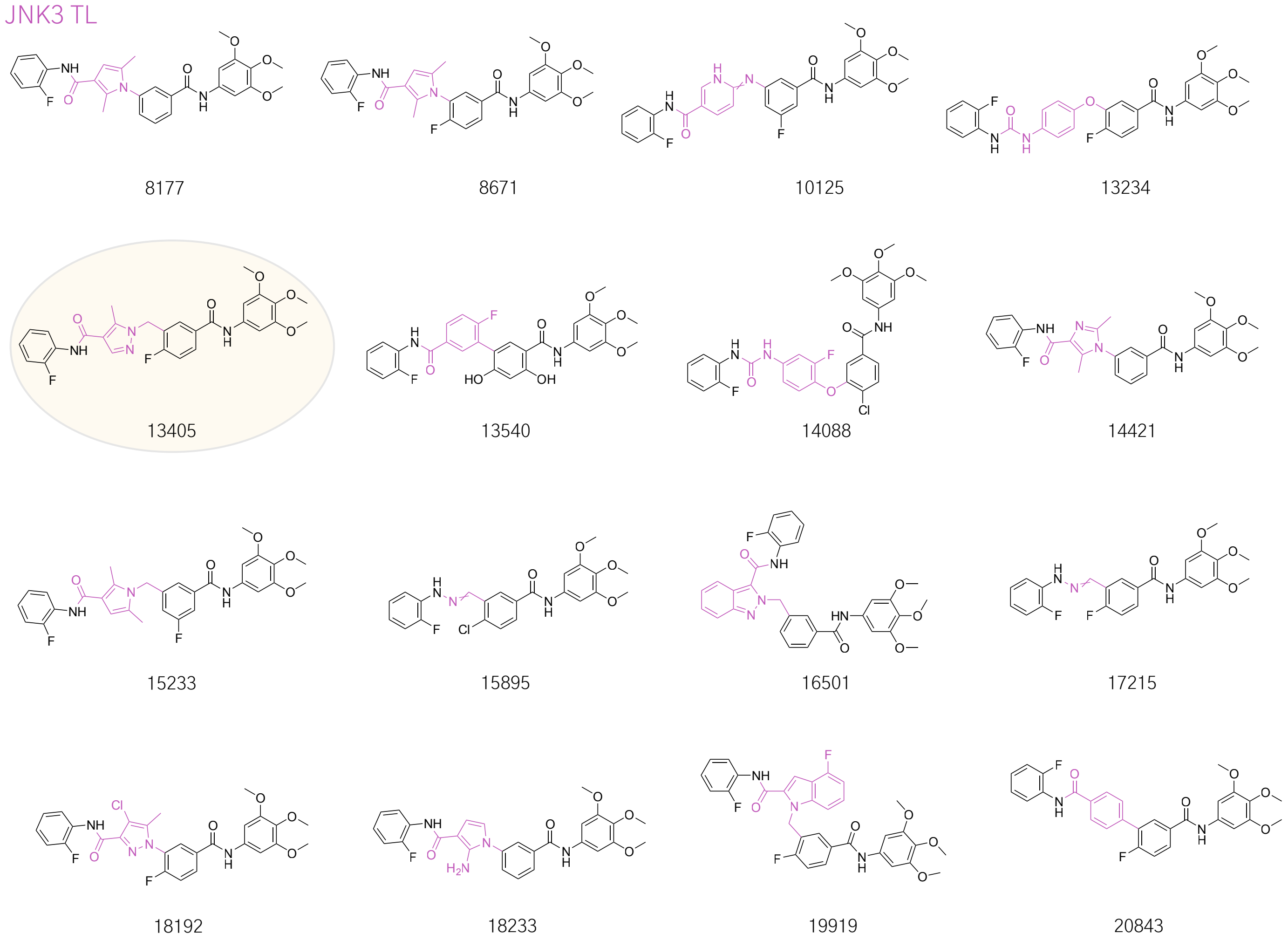

Fig. S5: 16 Highest scoring JNK3 TL designs around the published hop SR-3451. Highlighted in yellow is the most similar structure.

TABLE S6: 16 Highest scoring JNK3 TL designs around the published hop. *Predicted values. Clearance, permeability and LogP models are Novartis global models trained on in-house data.

**ID**	**score**	**epoch**	**clear. Human* [uL/min/mg]**	**perm. LE* [cm-6/s]**	**perm. PAMPA* [cm/s]**	**LogP***	**Severity Score*** [59]	**MolSkill*** [60]
8177	0.69	594	inconcl.	> 5	$>$ -4.5	4.1	0	3.6
8671	0.69	527	inconcl.	> 5	$>$ -4.5	4.2	0	4.2
10125	0.68	625	≤ 100	> 5	inconcl.	3.6	0	5.1
13234	0.67	321	≤ 100	> 5	inconcl.	4.3	0	6.1
**13405**	**0.67**	**1064**	**inconcl.**	**> 5**	**$>$ -4.5**	**3.5**	**0**	**4.5**
13540	0.67	552	≤ 100	> 5	inconcl.	4.1	1	10.3
14088	0.67	499	≤ 100	inconcl.	inconcl.	4.7	0	-0.3
14421	0.67	531	≤ 100	> 5	$>$ -4.5	3.7	0	1.4
15233	0.66	888	inconcl.	> 5	$>$ -4.5	4.0	0	2.2
15895	0.66	1284	inconcl.	> 5	$>$ -4.5	4.3	1	10.7
16501	0.66	575	inconcl.	> 5	$>$ -4.5	4.2	0	0.4
17215	0.66	797	≤ 100	> 5	$>$ -4.5	4.0	1	9.8
18192	0.65	348	≤ 100	> 5	$>$ -4.5	4.3	0	-2.8
18233	0.65	658	≤ 100	> 5	$>$ -4.5	3.6	0	0.5
19919	0.65	391	≤ 100	inconcl.	inconcl.	4.6	0	6.9
20843	0.64	302	≤ 100	inconcl.	inconcl.	4.4	0	3.8

4) ADCY10 TL

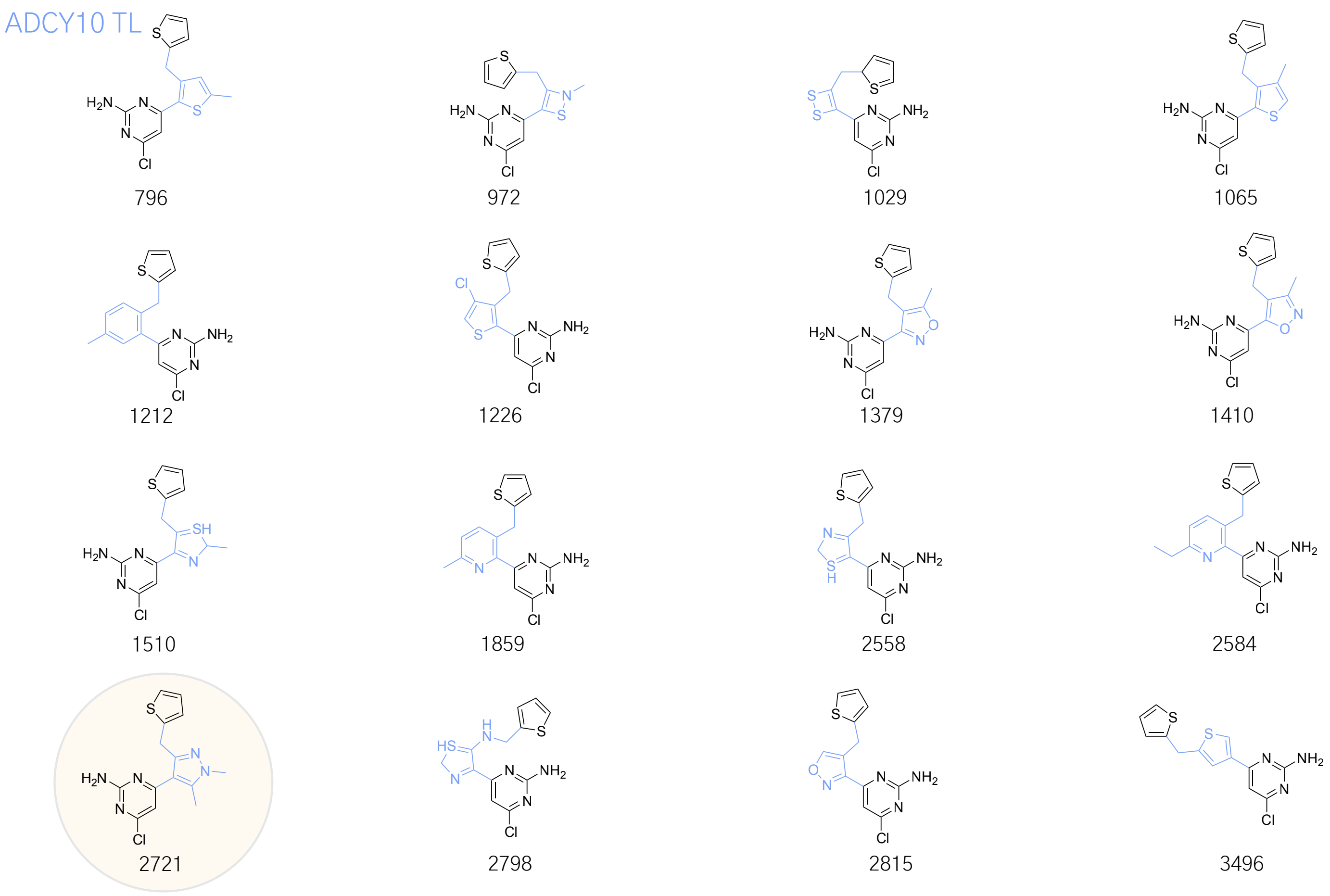

Fig. S6: 16 Highest scoring ADCY10 TL designs around the published hop TDI 09066. Highlighted in yellow is the most similar structure.

TABLE S7: 16 Highest scoring ADCY10 TL designs around the published hop. *Predicted values. Clearance, permeability and LogP models are Novartis global models trained on in-house data.

**ID**	**score**	**epoch**	**clear. Human* [uL/min/mg]**	**perm. LE* [cm-6/s]**	**perm. PAMPA* [cm/s]**	**LogP***	**Severity Score*** [59]	**MolSkill*** [60]
796	0.85	1248	inconcl.	> 5	> -4.5	4.2	0	-2.8
972	0.85	1338	inconcl.	> 5	> -4.5	3.7	0	-10.1
1029	0.84	1106	inconcl.	> 5	> -4.5	4.5	10	-4.2
1065	0.84	975	inconcl.	> 5	> -4.5	4.3	0	-1.7
1212	0.84	983	inconcl.	> 5	> -4.5	4.1	0	-2.4
1226	0.83	917	inconcl.	> 5	> -4.5	4.4	0	-2.0
1379	0.83	1189	inconcl.	> 5	> -4.5	3.3	0	-9.1
1410	0.83	956	inconcl.	> 5	> -4.5	3.3	0	-6.6
1510	0.82	1126	inconcl.	> 5	> -4.5	3.4	0	-4.6
1859	0.81	1115	inconcl.	> 5	> -4.5	3.4	0	-6.3
2558	0.79	1238	inconcl.	> 5	> -4.5	3.2	0	-3.1
2584	0.79	942	inconcl.	> 5	> -4.5	3.9	0	-8.5
**2721**	**0.78**	**1837**	**inconcl.**	**> 5**	**> -4.5**	**2.7**	**0**	**-8.6**
2798	0.78	1817	$\leq$ 100	> 5	> -4.5	3.6	0	0.5
2815	0.78	867	inconcl.	> 5	> -4.5	3.1	0	-6.4
3496	0.75	1745	inconcl.	> 5	> -4.5	4.3	0	-4.7

G. Discovery of New Scaffold-Hops

In this section, details about the post-processing are shown for each case study. We illustrate where high quality designs are generated (Supplementary Fig. 7, left hand plots), and how the generative chemical space is populated (Fig. 7, right hand plots).

The filter threshold for postprocessing are shown in Table S9 and S12. The exact filtering statistics for the full distributions are shown in Table S10, while the results for the control experiments are shown in Table S11, on which the thresholds are based. The scoring statistics of the selected post-processed designs are shown in Table S8.

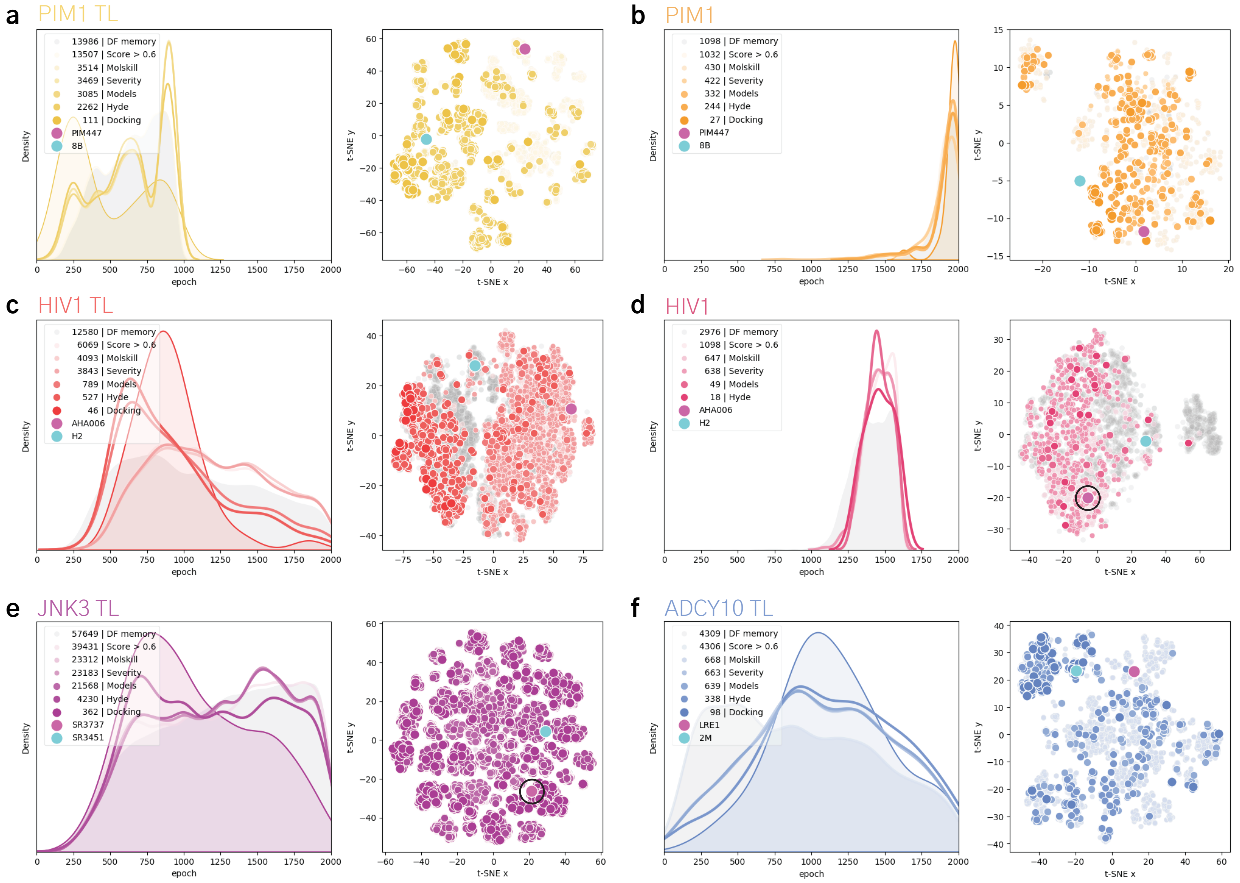

Fig. S7: **Post-processing and chemical space of *de novo* designs** produced by the reinforcement learning experiments. Shown on the left are the normalized distributions of designs that remained after every post-processing step. The threshold for these steps are reported in Supporting Table S9. The pre-filtered distributions are shown in grey. The post-processed set is shown in fill color. On the right are shown the *t*-distributed stochastic neighbour embeddings (t-SNE) (2 components, 100 perplexity, 42 random seed) [42] on ECFP6s (2048 bits, radius 3 [56]) of the same *de novo* designs. The points are colored (and sized) by the last processing step before removal. Each t-SNE was computed separately per experiment, with the reference and ground truth molecules included.

TABLE S8: Scoring statistics for selected designs. Reported are the epoch at which the design was first generated during reinforcement learning, their corresponding scoring function reward and Glide docking scores [43] obtained from post-processing.

Target	Design	Epoch	Scoring Function	Docking Score
PIM1	**12**	680	0.66	-10.1
	**13**	866	0.74	-10.7
	**14**	874	0.62	-10.4
	**15**	916	0.66	-10.2
	**16**	1961	0.68	-10.5
average			0.67 ± 0.04	-10.4 ± 0.24
HIV1	**17**	905	0.67	-11.0
	**18**	1049	0.64	-12.3
	**19**	712	0.66	-10.4
	**20**	1414	0.65	-10.2
average			0.66 ± 0.01	-11.0 ± 0.95
JNK3	**21**	1532	0.69	-11.5
	**22**	1054	0.63	-10.5
	**23**	641	0.68	-11.4
	**24**	417	0.68	-10.7
average			0.67 ± 0.03	-11.0 ± 0.50
ADCY10	**25**	1169	0.88	-12.5
	**26**	1115	0.81	-12.1
	**27**	377	0.85	-10.6
	**28**	1105	0.77	-10.4
	**29**	1376	0.85	-12.6
average			0.83 ± 0.04	-11.6 ± 1.06

H. Molecular Property Filters

Thresholds were based on in-house expertise and Lipinski's rule of five [61] (Table S9). The threshold for MolSkill was based on the authors' results for the ChEMBL dataset [60]. The threshold for HIV1 and JNK3 were adjusted according to the predicted values for the reference molecules **3** & **6**, as they fell significantly outside of this distribution (21.2 & 7.5 respectively). HIV1 reference molecules **3** & **4** were predicted to have a clearance above 300 $uL/min/mg$. For consistency, and in an effort to yield more chemically relevant designs, we opted to remain consistent with the clearance thresholds for the HIV1 designs.

TABLE S9: Post-processing property prediction thresholds for generated designs before docking. *20 for HIV1, 5 for JNK3.

Method	Threshold	Unit
SF reward	$<$ 0.6	-
clearance Human	$>$ 300	[uL/min/mg]
permeability LE	$\leq$ 1.5	[cm-6/s]
permeability PAMPA	$\leq$ -5.3	[cm/s]
Log P	$>$ 5	-
Severity Score [59]	$>$ 1	-
MolSkill [60]	$>$-5, 5, 20*	-

TABLE S10: **Post-processing of the de-novo designs**. For each target and each approach, we considered the designs that were stored by the diversity filter memory at the end of the reinforcement learning campaign. In other words, all unique designs that scored higher than 0.4, such that only designs that contain all decorations are considered. Molecules were filtered based on several predicted properties relevant for drug discovery (Supporting Table S9). The number of designs and the number and percentage of retained molecules are reported. ChEMBL = Pre-trained agent, FT = fine-tuning (on the reference bioactive). The highest number of generated and retained compounds per target is highlighted in boldface.

Target	Agent Training	Generated	Retained (%)
PIM1	ChEMBL Prior	1,098	27 (2.5%)
	ChEMBL + FT	**13,986**	**111** (0.8%)
HIV1	ChEMBL Prior	2,976	0 (0.0%)
	ChEMBL + FT	**12,580**	**46** (0.4%)
JNK3	ChEMBL Prior	0	-
	ChEMBL + FT	**57,649**	**362** (0.6%)
ADCY10	ChEMBL Prior	0	-
	ChEMBL + FT	**4,309**	**98** (2.3%)

I. Molecular Docking

TABLE S11: Predicted and computed statistics for the reference molecules and their published hops. Values that would be filtered out by the selected thresholds are highlighted in **boldface**. *Predicted values. Clearance, permeability and LogP models are Novartis global models trained on in-house data. **Values shown are the average scores of all stereo-isomers generated and docked by Glide, Table S12 & S1 reports the final thresholds used in post-processing.

Target	ID	clear. Human*	perm. LE*	perm. PAMPA*	LogP*	Severity Score*	Mol Skill*	rot. bonds	stereo centers	mol. weight	DOCKING SCORE	INTERNAL ENERGY	INTRA CLASH	TORSION QUALITY
PIM1	PIM447**	$\leq$ 100	>5	>-4.5	3.7	0	-12.0	4	3	440.5	-10.4	3.9	green	green
	8B	$\leq$ 100	>5	>-4.5	3.1	0	-6.7	4	1	421.5	-10.4	1.9	green	green
HIV1	AHA006**	**>300**	>5	>-4.5	4.4	0	**21.2**	10	4	574.7	-10.6	**21.6**	green	**red**
	H2**	**>300**	>5	>-4.5	4.0	0	**11.6**	15	3	561.6	-11.1	**18.0**	green	**red**
JNK3	SR3737	$\leq$ 100	inconcl.	inconcl.	4.7	0	**3.8**	8	0	512.5	-13.4	**10.0**	green	green
	SR3451	$\leq$ 100	>5	inconcl.	3.9	0	**7.5**	8	0	487.5	-10.2	**6.4**	green	yellow
ADCY 10	LRE1	$\leq$ 100	>5	>-4.5	4.0	0	-4.1	4	0	280.8	-11.4	2.7	green	yellow
	TDI9066	$\leq$ 100	>5	>-4.5	2.7	0	-7.5	3	0	305.8	-12.6	0.2	green	green

TABLE S12: Post-processing thresholds for retention of generated samples after docking. See Supporting Table S11 self docking results of the reference molecules.

Method	Metric	Threshold	HIV1	JNK3
Glide	INTERNAL ENERGY	< 2	< 15	< 5
	DOCKING SCORE	< -10		
Hyde	TORSION QUALITY	green		
	INTRA CLASH	green	! red	

III. Benchmarking

This section contains additional details on the benchmarking experiments. We show the complete distributions of ROCS Shape and Color scores for the benchmarked models in Fig. S8.

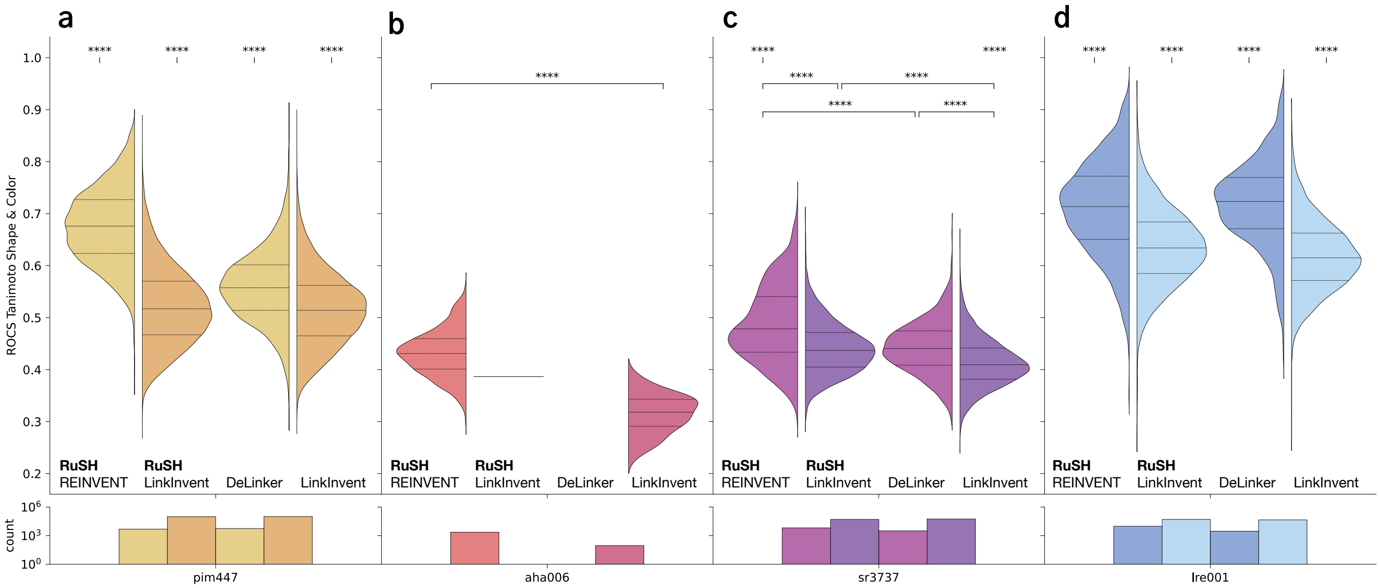

Fig. S8: Distributions of ROCS Tanimoto Shape & Color scores for generated designs in the benchmark analysis, Table 1. From left to right, results are shown with respect to reference ligands (**a**) PIM 447, (**b**) AHA 006, (**c**) SR-3737, and (**d**) LRE 1. Results are ordered from left to right, RuSH (REINVENT), RuSH (LinkInvent), DeLinker, LinkInvent. Distribution sample size is plotted directly below, in log scale. A Kruskal-Wallis H-test was performed per case study, followed by post-hoc Dunn to test for statistical significance with Bonferroni correction if a p-value < 0.05 was found. **** $p < 0.0001$.

A. *LinkInvent*

This section contains the scoring function parameters for the default LinkInvent configuration, used during benchmarking.

TABLE S13: Scoring function components and parameters for the Link-INVENT reinforcement learning experiments, based on properties found in the reference molecules.

Target	Molecular Weight.	No. H-bond Donors	No. H-bond Acceptors	No. Rings
PIM1	76 – 200	0 – 1		0 – 2
HIV1	320 – 444	0 – 2	0 – 2	2 – 4
JNK3	54 – 178		0 – 1	0 – 2
ADCY10	14 – 130			0 – 2

IV. Conformer Generation

This section shows preliminary results for the number of conformers generated during scoring.

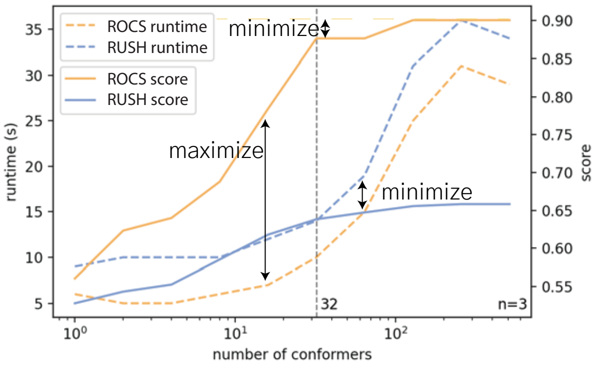

Fig. S9: Preliminary results for number of conformers vs runtime (s) plotted for ROCS and RUSH. The chosen the number of conformers for reinforcement learning experiments was selected to minimize runtime and maximize score quality with respect to the control.

V. Effect of allowance (α) on reinforcement learning

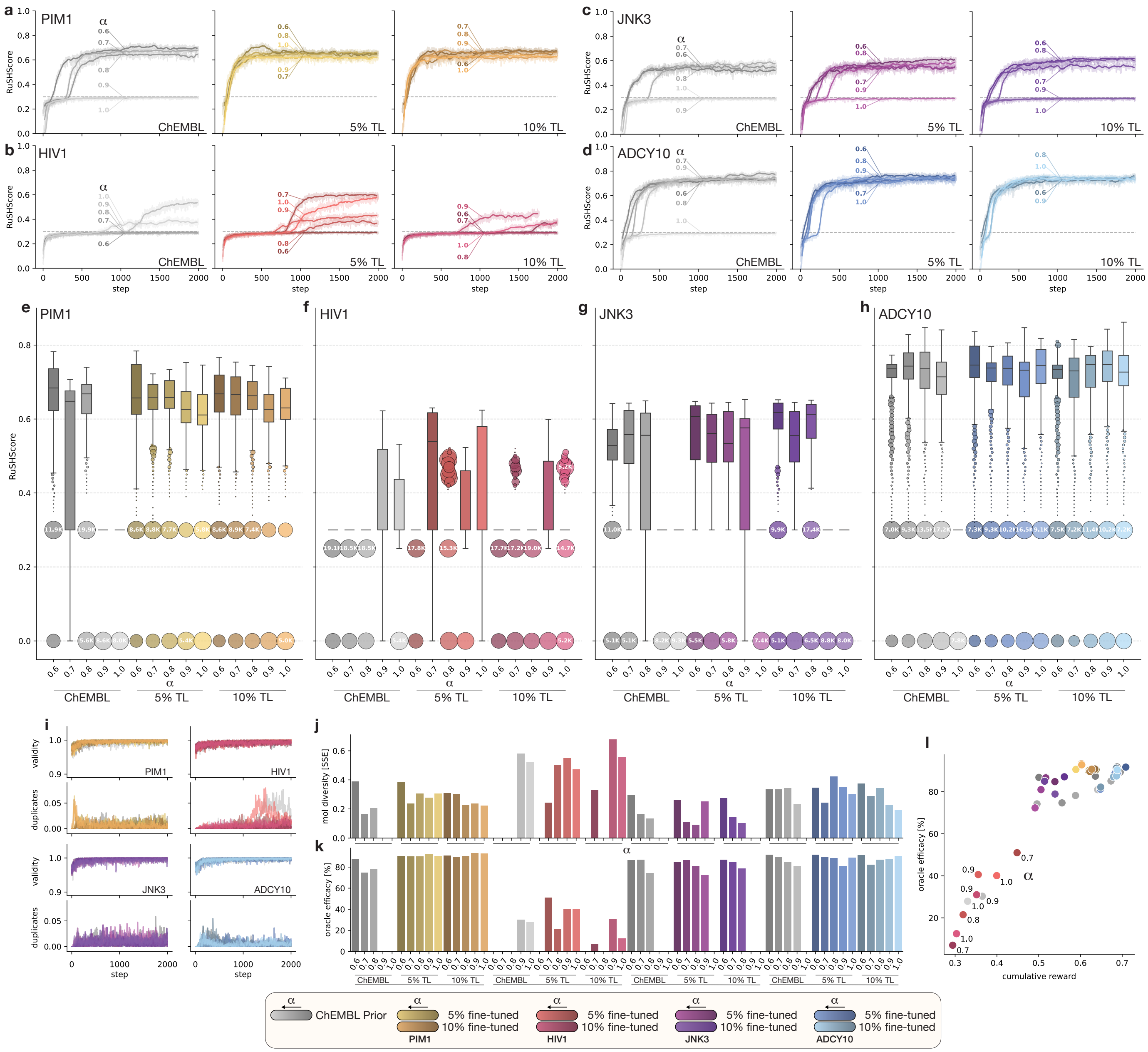

Fig. S10: **Effect of allowance (α) on reinforcement learning.** (**a-d**) Learning behavior for (**a**) PIM1, (**b**) HIV1, (**c**) JNK3, and (**d**) ADCY10. We vary the allowance parameter (α) from 0.6 to 1.0 at 0.1 intervals. Experiments were split between 3 prior models using REINVENT4: 1) A prior trained on ChEMBL34 [21] using default parameters [30]; 2) That same prior fine-tuned with the reference ligand (**1**,**3**,**5**,**7**) for 5% sampling bias (Eq. S2); 3) and 10% sampling bias. This resulted in 15 experiments per case study. RL was done for 2000 epochs, 64 batch size. Performance was consistent across α per case-study. An increase in α (primarily $\alpha = 1.0$) can lead to difficulty learning the inclusion. This behavior can be easily resolved with fine-tuning. α shows no effect on score convergence or cumulative reward for cases that learned the inclusion. (**e-f**) Scoring statistics for generated designs during RL. Outliers are shown as circles, with size proportional to the number of outliers. Scoring performance depends primarily on the case-study. We observe a sizable portion of designs are spent learning the inclusion, and can be seen as outliers at $y = 0.0$ and 3.0 (0.25 for HIV1. See Eq. 1). As α increases, the number of these outliers tends to increase. This is expected, as inclusion criteria become more strict. (**i**) Validity and duplicates (inverse of uniqueness) of sampled designs during RL. We observe stable validity and uniqueness across all experiments, showing α is not destructive to the model's generative performance. (**j**) Molecular diversity, measured as Scaled Shannon Entropy [SSE] (Eq. S1). Diversity was computed on the set of designs that scored above 0.4, to only consider designs with correct decoration inclusion. We do not observe clear trends on diversity as a function of α. Rather, results depend mostly on the case-study and coincide with findings from Figure 5. (**k**) Oracle efficacy, defined as the number of designs that scored above 0.4 ("succeeded") divided by the total number of generated designs. We see consistent efficacy across α. For all cases except HIV1, greater than 70% off generated designs successfully included the reference decorations and scored above 0.4. (**l**) Oracle efficacy as a function of cumulative reward (normalized by the number of epochs). All experiment performed well, except HIV1. These are annotated with α, and show no correlation between α and performance. These findings illustrate robust performance of allowance (α) across experiments, providing the user with dynamic tuning of decoration inclusion without detriment to model performance.
